# Multiscale comparative connectomics

**DOI:** 10.1162/IMAG.a.2

**Published:** 2025-05-16

**Authors:** Vivek Gopalakrishnan, Jaewon Chung, Eric Bridgeford, Benjamin D. Pedigo, Jesús Arroyo, Lucy Upchurch, G. Allan Johnson, Nian Wang, Youngser Park, Carey E. Priebe, Joshua T. Vogelstein

**Affiliations:** Department of Biomedical Engineering, Johns Hopkins University, Baltimore, MD, United States; Department of Biostatistics, Johns Hopkins University, Baltimore, MD, United States; Department of Statistics, Texas A&M University, College Station, TX, United States; Center for In Vivo Microscopy, Department of Radiology, Duke University, Durham, NC, United States; Department of Biomedical Engineering, Duke University, Durham, NC, United States; Department of Radiology and Imaging Sciences, Indiana University School of Medicine, Indianapolis, IN, United States; Center for Imaging Science, Johns Hopkins University, Baltimore, MD, United States; Department of Applied Mathematics and Statistics, Johns Hopkins University, Baltimore, MD, United States

**Keywords:** statistical connectomics, random graph models, network neuroscience

## Abstract

The connectome, a map of the structural and/or functional connections in the brain, provides a complex representation of the neurobiological phenotypes on which it supervenes. This information-rich data modality has the potential to transform our understanding of the relationship between patterns in brain connectivity and neurological processes, disorders, and diseases. However, existing computational techniques used to analyze connectomes are often insufficient for interrogating multi-subject connectomics datasets: many current methods are either solely designed to analyze single connectomes or leverage heuristic graph statistics that are unable to capture the complete topology of multiscale connections between brain regions. To enable more rigorous connectomics analysis, we introduce a set of robust and interpretable statistical hypothesis tests motivated by recent theoretical advances in random graph models. These tests facilitate simultaneous analysis of multiple connectomes across different scales of network topology, enabling the robust and reproducible discovery of hierarchical brain structures that vary in relation to phenotypic profiles. In addition to explaining the theoretical foundations and guarantees of our algorithms, we demonstrate their superiority over current state-of-the-art connectomics methods through extensive simulation studies and real-data experiments. Using a set of high-resolution connectomes obtained from genetically distinct mouse strains (including the BTBR mouse—a standard model of autism—and three behavioral wild-types), we illustrate how our methods successfully uncover latent information in multi-subject connectomics data and yield valuable insights into the connective correlates of neurological phenotypes that other methods do not capture. The data and code necessary to reproduce the analyses, simulations, and figures presented in this work are available athttps://github.com/neurodata/MCC.

## 1 Introduction

Understanding how patterns in brain connectivity give rise to observable biological phenotypes is a central pursuit in neuroscience. Derived from neuroimaging data, the connectome (a graphical representation of neural connections) has recently become an invaluable modality for such analyses, allowing researchers to represent organisms’ brains as networks and understand nervous system organization with graph theoretical methods (Bullmore & Sporns, 2009). Successfully associating biological phenotypes with organizational variation in the connectome will enable the identification of neurological structures and circuits that drive cognitive function (Bullmore & Bassett, 2011). However, to fully realize the promise of the connectome, new statistical graph theory methods that are principled, robust, and reproducible are required for the analysis of this nascent and highly-complex datatype (Craddock et al., 2013).

From a mathematical perspective, a connectome can be modeled as a network (also referred to as a graph) of the interactions between brain regions (Sporns et al., 2005). In this network, vertices represent disjoint regions of the brain, and edges represent the connections between these regions (Vogelstein et al., 2019). From a neuroscientific perspective, the connectome can be further described at multiple hierarchical scales of network topology (Kaiser, 2011): at the extremes are the*local scale*, characterized by the features of individual edges and vertices in the connectome, and the*global scale*, characterized by patterns in brain connectivity that are often quantified by a variety of graph statistics (Rubinov & Sporns, 2010); the intermediate*regional scale*focuses on the interactions between distinct subsets of brain regions (known as communities or blocks), comprising subgraphs of the connectome (Vogelstein et al., 2013;S. Wang et al, 2018). Simultaneously considering the local, regional, and global scales of network topology provides a multiscale view of patterns in neural connectivity.

The fundamental goal of multi-subject connectomics is to identify the multiscale patterns in brain network architecture that differ across phenotypic groups (van den Heuvel et al, 2016). However, given the inherent structure of the connectome, special care needs to be taken when designing analytical methods. The naive application of classical statistical tests to network-valued data can sometimes produce misleading results. For example, many methods use graph statistics (e.g., clustering coefficient, degree centrality, etc.) to characterize differences in the connectivity patterns within connectomes; however, recent studies have shown that no set of graph statistics can comprehensively describe network topology, as networks with wildly different structures can produce identical graph statistics (Chen et al., 2019;Chung et al., 2021). That is, the one-number summaries of edge-, vertex-, and community-scale connectivity provided by graph statistics often fail to comprehensively describe neurological topology. This is not to say the graph statistics are not a useful analytic tool (they can be intuitive and logical descriptors of individual connectomes), but rather that they are often insufficient when building statistical tests for comparative connectomics. In multiple simulation studies and real-data experiments, we demonstrate that tests based on graph statistics do not always perform accurate inference, failing to recapitulate known neurological phenomena from connectomes.

**Fig. 1.**
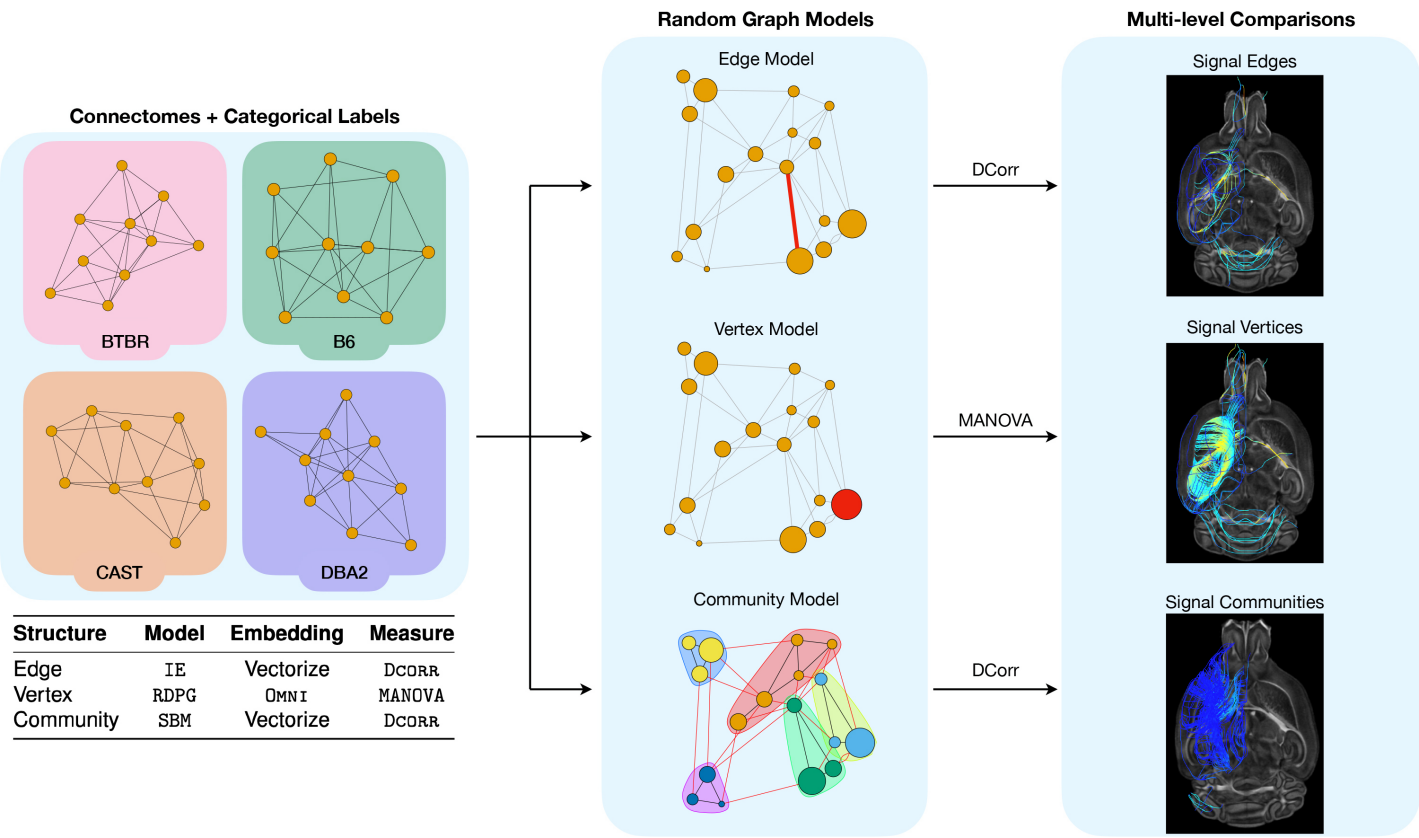
Overview of the statistical framework for multiscale inference in comparative connectomics, summarizing the random graph model, embedding method, and statistical test used for edges, vertices, and communities, respectively. Left: All connectomes are defined on a common set of vertices and have an associated categorical label. Illustrative networks from four mouse lines whose connectomes we analyze in this work are shown. Center: Connectomes are fit to random graph models that are specifically chosen to model the variation in a network at a given topological scale. Right: Using the categorical labels,k-sample statistical hypothesis tests are applied to the estimated parameters from each model, yielding a set of the signal edges, vertices, and communities across the groups in a given multi-subject connectomics dataset. Finally, we visualize the strongest signal component at each scale using tractography.

To overcome these limitations and enable the rigorous interrogation of multi-subject connectomics datasets, we present a set of network-based statistical hypothesis tests that provide insight across topological scales of the connectome (Fig. 1). Predicated on recent advances in the theory of random graph models, our methods can be used to identify neurobiological structures within connectomes that are connectively different across multiple categorical or dimensional phenotypes, which we term*signal*components. Specifically, these tests can be used to discover signal edges, vertices, and communities within populations of connectomes defined on the same vertex set and are appropriate for analyzing connectomes estimated from either structural or functional neuroimaging data. We formulate these methods ask-sample hypothesis tests, enabling comparisons of connectomes from more than two distinct phenotypic groups. Finally, we show how the effect sizes measured by our tests can be aggregated across scales, helping to overcome the limitations of multiple hypothesis testing that is inherent to high-dimensional connectomics data.

Modeling connectomes with random graph models allows us to mathematically characterize patterns in network structure, as well as to account for noise within and across samples. Such models have many interpretable and provable properties, which we leverage to formulate principled statistical tests for multi-subject connectomics data. We demonstrate the efficacy and utility of this connectomics paradigm by applying our multiscale tests to an open access dataset of ultrahigh-resolution structural mouse connectomes derived at a spatial resolution 20,000 times greater than typical human connectomes (N. Wang et al., 2020). Additionally, we compare the performance of our proposed tests to prevailing connectomics analysis strategies in extensive simulation studies, and show that the neurobiological insights of our measures of connectivity are orthogonal to existing measures through information-theoretic comparisons. Taken together, our proposed methods enable principled interrogation of the local, regional, and global scales of network topology, providing researchers with novel tools for testing neurobiological hypotheses in multi-subject connectomics datasets.

## 2 Preliminaries

### 2.1 Graph theory notation

Networks (or graphs) are convenient mathematical objects for representing connectomes. A networkGconsists of the ordered pair(V,E), whereVis the set of vertices andEis the set of edges. The set of vertices can be represented asV={1,2,…,n}, where|V|=nis the number of vertices. The set of edges is a subset of all possible connections between vertices (i.e.,E⊆V×V). We say the tuple(i,j)∈Eif there exists an connection between vertexiand vertexj. In many connectomics datasets, edges have associated edge weights, that is, real-valued numbers that encode quantitative information about a connection between two vertices. The interpretation of the edge weight is dependent on the imaging modality used to measure the connectome. For example, the edge weights in structural connectomes are non-negative integers that represent the number of neuronal fibers that traverse from one region of the brain to another (Kiar et al., 2017). Every connectome has an associated weighted adjacency matrix$A\in {\mathbb{R}}^{n\times n}$where$A_{ij}$denotes the weight of the edge(i,j)∈E.

### 2.2 Random graph models

Statistical modeling of connectomics data enables the principled analysis of high-dimensional, graph-valued data. Random graph models treat individual connectomes as random variables, enabling mathematical characterization of network structure and accounting for noise within and across observed samples. In the following paragraphs, we present the three random graph models we use to construct our multiscale tests: 1) the Independent Edge (IE) model; 2) the Random Dot Product Graph (RDPG); and 3) the Stochastic Block Model (SBM). Each model is designed to characterize a particular topological scale of the connectome (i.e., either its edges, vertices, or communities). Treating connectomes as random network-valued variables sampled from these random graph models enables the formulation ofk-sample hypothesis tests that can be used to identify connective differences at multiple scales across numerous phenotypic profiles.

*The edge model.*In the Independent Edge (IE) model, every possible edge(i,j)∈V×Vis sampled from independent Bernoulli distributions, each parameterized by some edge-specific probability$p_{ij}\in [0,1]$. These can be summarized using a matrix of edge-wise probabilities$\text{P}\in {\left[0,1\right]}^{n\times n}$, where${\text{P}}_{ij}=p_{ij}$. In our formulation of the edge-wise hypothesis test (§3.1), we consider the weighted IE model to account for a network with weighted edges. For this variant, instead of treating each edge as a Bernoulli random variable, we sample each edge weight from a distribution$F_{ij}$supported on the set of non-negative real numbers. In the weighted IE model,Prepresents a matrix of univariate probability distributions modeling the weight of each edge in the connectome. We say a network is sampled from the model, that is,G∼IE(P), if its adjacency matrixAhas entries${\text{A}}_{ij}∼F_{ij}$independently for every edge in the connectome. When estimating thePmatrix for a weighted IE model, we assume that all elements ofPare from the same family of distributions.

*The vertex model.*The Random Dot Product Graph (RDPG) is a member of the family of latent position random graphs, a class of models where the probability of a connection$p_{ij}$is determined by the vertices, not the edges (Hoff et al., 2002). Under such models, each vertexi∈Vis associated with a*latent position*$x_{i}$, which belongs to some*latent space*X. The probability of a connection between verticesiandjis given by a link functionκ:X×X↦[0,1]; that is,$p_{ij}=\kappa (x_{i},x_{j})$. In the RDPG, the latent spaceXis a subspace of Euclidean space${\mathbb{R}}^{d}$and the link function is the dot product (Scheinerman & Tucker, 2010). Thus, in ad-dimensional RDPG withnvertices, the rows of the matrix$\text{X}\in {\mathbb{R}}^{n\times d}$encode the latent position of each vertex, and the matrix of edge-wise connection probabilities is given by$\text{P}=\text{X}{\text{X}}^{⊺}$. A network is sampled from the modelG∼RDPG(X)if its adjacency matrixAhas entries${\text{A}}_{ij}∼\text{Bernoulli}(x_{i}\cdot x_{j})$independently for every edge(i,j)∈V×V.

The modeling assumptions of the RDPG make estimation of the latent positions analytically tractable. The estimation procedure we use is the omnibus embedding (Omni) (Athreya et al., 2017), which jointly estimates the latent positions for every connectome in a dataset by mapping each vertex to a vector in${\mathbb{R}}^{d}$that corresponds to the vertex’s latent position in ad-dimensional RDPG. A host of downstream machine-learning tasks can be accomplished with this jointly embedded representation of a sample of connectomes, such as clustering or classification of vertices. Here, we use the embedding to formulate a hypothesis test that can be used to identify vertices that are strongly associated with given phenotypes. Note thatOmniis a member of a larger class of*vertex embedding*algorithms, that is, algorithms that map vertices to real-valued vectors. However, the vectors produced by other vertex embedding algorithms do not correspond to parameters in an RDPG. We compareOmnito many other vertex embedding methods, including vertex-level graph statistics, in the Results. Our experiments demonstrate that the embeddings produced byOmnienable accurate statistical inference by identifying signal vertices with known neurobiological significance, whereas previously proposed vertex embedding methods do not.

*The community model.*In the Stochastic Block Model (SBM), every vertex belongs exclusively to one ofKcommunities, which partition the vertex set (Holland et al., 1983). The SBM is a special case of the RDPG in which all vertices from the same community have identical latent positions; that is, the connection probability is solely determined by community membership. A symmetricK×Kcommunity connectivity probability matrixBwith entries in${\left[0,1\right]}^{K\times K}$governs the probability of an edge between two vertices given their community memberships. Community membership is determined by a vertex assignment vector$\vec{\tau }\in {\left\{1,\ldots ,K\right\}}^{n}$, which is either estimated from the data (Newman, 2004;Que et al., 2015) or given*a priori*. As brain regions in different atlases can be hierarchically grouped into superstructures and hemispheres, we assume that$\vec{\tau }$is given. Thus, a network is sampled from the model,G∼SBM(B)with$\vec{\tau }$given, if its adjacency matrixAhas entries${\text{A}}_{ij}∼\text{Bernoulli}({\text{B}}_{k_{i}k_{j}})$where$\tau_{i}\;=k_{i}$and$\tau_{j}=k_{j}$for(i,j)∈V×V, and$k_{i},k_{j}\in \left\{1,\ldots ,K\right\}$. As with the IE model, a weighted variant of the SBM can be constructed by replacing the block probability${\text{B}}_{ij}$with a probability distribution$F_{ij}$for the entire block.

## 3 Methods

### 3.1 Multiscale inference

We introduce ourk-sample hypothesis tests using a formal statistical framework. As the random graph models we use to formulate these tests do not assume a specific statistical distribution at each scale of network connectivity, our methods are applicable to structural and functional connectomes, as well as directed and undirected graphs. Practical demonstrations of how our methods can be used to analyze real-world connectomics data are provided in the Results (§4).

#### 3.1.1 Identifying signal edges

The simplest approach for comparing connectomes is to treat them as a*bag of edges*without considering interactions between the edges (Craddock et al., 2013). Serially performing univariate statistical tests at each edge enables the discovery of*signal edges*whose neurological connectivity differs across categorical or dimensional phenotypes. Using the IE model, we assume that each connectome is sampled from a phenotype-conditional probability matrix; that is, we assume that for each phenotype in$Y=\{c_{1},\ldots ,c_{k}\}$, there is an associated probability matrix in$\left\{{\text{P}}^{c_{1}},\ldots ,{\text{P}}^{c_{k}}\right\}$from which all connectomes in that phenotype are sampled. For a given edge(i,j), we assume the edge weight for each connectome has been independently and identically (i.i.d) sampled from the appropriatePmatrix. Specifically, we assume that for every connectome in phenotype$c_{y}\in Y$, the edge weight${\text{A}}_{ij}∼{\text{P}}_{ij}^{c_{y}}$i.i.d. Following this assumption, we formulate the following null and alternative hypotheses:

$$\begin{array}{l} H_{0}:\forall \left(y,y^{\prime }\right):{\text{P}}_{ij}^{c_{y}}={\text{P}}_{ij}^{c_{y^{\prime }}}\\ H_{1}:\exists \left(y,y^{\prime }\right):{\text{P}}_{ij}^{c_{y}}\neq {\text{P}}_{ij}^{c_{y^{\prime }}} \end{array}$$ (1)

That is, the null hypothesis is that weight distribution for a particular edge(i,j)is identical across all phenotypic groups, whereas the alternative hypothesis is that this distribution is different for at least one of the phenotypes.

To test the null hypothesis, any univariate statistical test can be employed since edge weights are themselves scalar random variables in the IE model. For this task, since the entries ofPare themselves distributions, we use Distance Correlation (Dcorr), a previously established universally consistent nonparametrick-sample test for equality in distribution (Panda et al., 2020;Székely et al., 2007). In the Supplement (§A.1), we demonstrate that Dcorris a more powerful test than commonly used non-parametric and Gaussian alternatives.

As a final consideration, note that serial edge-wise testing requires corrections for an immense number of multiple comparisons. If the sample consists of directed connectomes, then the total number of tests is$n^{2}$; if the connectomes are undirected, then the total number of tests is$\left(\begin{array}{c} n\\ 2 \end{array}\right)$.

#### 3.1.2 Identifying signal vertices

We test for differences in a given vertex’s connectivity by comparing its latent position estimates fromOmniacross phenotypes. According to a Central Limit Theorem forOmni, these latent position estimates are universally consistent and asymptotically normal (Levin et al., 2017). This motivates our use of normal-theory inferential statistical tests to determine if the embedding of a given vertex is different across phenotypes. If the number of classesk=2, we use Hotelling’s T-squared (Hotelling), a multivariate generalization of thet-test; ifk>2, we use one-way MANOVA with Pillai’s trace as our test statistic. We formulate the following null and alternative hypotheses:

$$\begin{array}{l} H_{0}:\forall \left(y,y^{\prime }\right):\mu_{i}^{c_{y}}=\mu_{i}^{c_{y^{\prime }}}\\ H_{1}:\exists \left(y,y^{\prime }\right):\mu_{i}^{c_{y}}\neq \mu_{i}^{c_{y^{\prime }}} \end{array}$$ (2)

where$\mu_{i}^{c_{y}}$is the mean latent position of the vertexifor the phenotype$c_{y}\in Y$. This procedure results in a total ofnstatistical tests, one for each vertex in the vertex setV.

#### 3.1.3 Identifying signal communities

Vertices in a connectome can be hierarchically organized into superstructures such as major brain regions and hemispheres. The interactions within and between these communities of vertices form connective circuits within the brain, and are more correlated with complex behavior and phenotypes than individual edges or vertices. Therefore, interrogation of the*regional scale*and identification of signal communities is a critical component of multiscale connectomics analysis.

Here, we use the SBM to model the community structure of a connectome. We propose four approaches for describing the connectivity of a community, and an accompanying statistical procedure for each approach. Each subsequent approach provides an increasingly more holistic description of a community. For a given block(i,j)in the SBM, and for each phenotype, we posit the following tests:

1. **Average Connectivity:**Compute the average number of nonzero edges (binarize using Otsu’s method (Otsu, 1979)), which is equivalent to the community connectivity probability${\text{B}}_{ij}$. We then use Pearson’s chi-squared to determine if there is a significant difference in the connectivity probability across phenotypes.
2. **Average Edge Weight:**Compute the average weight of all the edges, essentially treating the community as a single large edge. We then use Dcorrto determine if there is a significant difference in the average edge weight across phenotypes.
3. **Multivariate Binary:**Binarize the community using Otsu’s method (Otsu, 1979), and vectorize the subgraph of the adjacency matrix corresponding to that community. We binarize to determine if the distribution of nonzero edges is different across phenotypes. Test for differences in this binary representation usingDcorr.
4. **Multivariate Weighted:**Vectorize the subgraph of the adjacency matrix corresponding to that community. Again, test for differences in this weighted representation usingDcorr.

If the connectomes in question haveKcommunities and are directed, this procedure results in$K^{2}$comparisons. If the connectomes are undirected, this procedure results in$\left(\begin{array}{c} K\\ 2 \end{array}\right)$comparisons.

### 3.2 Correcting for multiple comparisons

Conventional atlases for multi-subject connectomics studies are typically composed of hundreds of vertices and tens of thousands of edges (Myers et al., 2019). Therefore, as demonstrated by the formulations in the Methods (§3.1), the multiscale statistical tests we introduce result in a large number of multiple comparisons. To limit the number of structures that are falsely identified as signal components, we consider methods for controlling the Family-Wise Error Rate (FWER), the probability of making at least one false discovery, and the False Discovery Rate (FDR), the proportion of discoveries expected to be false.

Well-established methods for controlling the FWER like the Bonferroni correction (Weisstein, 2004) are amenable to applications in connectomics because they do not assume that the multiple hypotheses are independent of each other. Such independence would be a logically impossible condition for all but the simplest random graph models (e.g., weighted IE) as each edge is defined specifically by virtue of a dependence between pairs of vertices. However, the stringent definition of FWER makes the Bonferroni correction (and even its uniformly more powerful extension, the Holm–Bonferroni correction (Holm, 1979)) overly conservative. By comparison, the FDR-controlling Benjamini–Hochberg correction is less conservative; however, to guarantee control of the FDR, the procedure assumes that the p-values from true null hypotheses (called*null p-values*) are mutually independent of one another, as well as being independent of the non-null p-values (Benjamini & Hochberg, 1995). When applied to connectomics data, which necessarily violates the independence assumption, Benjamini–Hochberg can result in overly liberal statistical corrections and higher rates of Type 1 Error (Efron, 2008). To err on the side of statistical caution, we use the Holm–Bonferroni correction as its lack of an independence assumption makes it valid for connectomics data. By adopting FWER control instead of FDR control, we greatly reduce the likelihood of our algorithms producing spurious results.

To ameliorate the conservative nature of FWER control, we leverage a unique advantage of our multiscale approach. Unlike single-scale methods for comparative connectomics, our proposed methods are able to*borrow power*across scales of the connectome. For example, given the relatively small sample sizes in connectomics datasets, it is difficult to accurately estimate the importance of a particular edge (tens of subjects versus hundreds of thousands of edges). However, it is more feasible to estimate the importance of a particular vertex (tens of subjects versus hundreds of vertices). Then, if a given vertex is determined to be a signal vertex, some subset of the incident edges must also be signal edges. This allows inferences at coarser resolutions of network connectivity to inform inferences at finder resolutions, where the dimensionality is so high that traditional statistical hypothesis tests will not be sufficiently powerful to identify signal components.

### 3.3 A multi-subject mouse connectome dataset for algorithmic validation

In the Results (§4), we provide illustrative examples of how our multiscale hypothesis tests operate on real-world data by applying them to an open access dataset of whole-brain diffusion magnetic resonance imaging-derived connectomes from four mouse lines: BTBR T+ Itpr3tf/J (BTBR), C57BL/6J (B6), CAST/EiJ (CAST), and DBA/2J (DBA2) (N. Wang et al., 2020). The BTBR mouse strain is a well-studied model that exhibits core behavioral deficits that characterize autism spectrum disorders (ASD) in humans (McFarlane et al., 2008;Scattoni, et al., 2008;Silverman et al., 2010). Additionally, the BTBR mouse has significant neuroanatomical abnormalities, including the complete absence of the corpus callosum, a band of nerve fibers connecting the left and right hemispheres of the brain (Ellegood et al., 2013;Meyza & Blanchard, 2017). Therefore, we can use this dataset to determine if our tests (as well as other existing connectomics methods) will successfully recover this previously established neurobiological information. The B6, CAST, and DBA2 mice are genetically distinct strains that do not exhibit ASD-like behaviors; they serve as wild-type behavioral controls in these experiments.

For each strain, connectomes were generated from eight age-matched mice (N=8per strain with a sex distribution of four males and four females) using diffusion tensor imaging (DTI) tractography as described in the Methods (§3.4). Improved imaging protocols and custom hardware were developed and implemented to solve existing technical issues with DTI, including an inability to resolve crossing and merging fibers; for more details, see §2.2 of (N. Wang et al., 2020). Each connectome was parcellated using a symmetric Waxholm Space (Calabrese et al., 2015;Johnson et al., 2010), yielding a vertex set with a total of 326 regions of interest (ROIs) bilaterally distributed across the left and right hemispheres, and an undirected edge set with 52,975 edges. Within a given hemisphere, there were seven superstructures consisting of multiple ROIs, resulting in a total of 14 distinct communities in each connectome. Heatmaps of the average log-transformed adjacency matrix for each strain with hierarchical community and hemispheric labels are shown inSupplementary Figure 1.

### 3.4 Tractography

To visualize the neurological structures identified by our methods, deterministic tractograms were generated in DSI Studio using the generalized Q-sampling fiber tracking algorithm (Yeh et al., 2013). For the specific parameter values used for tract generation, see §2.6 ofN. Wang et al. (2020). A group average template was constructed from the four male mice per strain. A DTI diffusion scheme was used, and a total of 46 diffusion sampling directions were acquired. The b-value was 4000 s/mm^2^. The in-plane resolution and the slice thickness were both 0.045 mm. The diffusion data were reconstructed in the MNI space using q-space diffeomorphic reconstruction (Yeh & Tseng, 2011) to obtain the spin distribution function (Yeh et al., 2010). A diffusion sampling length ratio of 1.25 was used. The restricted diffusion was quantified using restricted diffusion imaging (Yeh et al., 2017).

## 4 Results

### 4.1 Interrogating the *local scale*

Illustrative applications of our proposed local-scale algorithms, along with comparisons to existing connectomics methods, are provided below. The mathematical formulations of these algorithms for identifying signal edges and signal vertices are provided in the Methods (§3.1).

#### 4.1.1 Identifying signal edges

Univariate edge-wise testing provides an interpretable and computationally tractable method for identifying connective differences in specific edges across phenotypes. This is a well-established analytical approach in connectomics, and many classical statistical tests like one-way analysis of variance (ANOVA) and the Kruskal–Wallis*H*test are commonly used for edge-wise testing (Craddock et al., 2013;Dagenbach, 2019;Varoquaux & Craddock, 2013). However, these are tests of equality in*location*: that is, ANOVA (Kruskal–Wallis) tests whether the mean (median) weight of a particular edge is statistically different across phenotypes. If the distribution of an edge weight is different across phenotypes but the mean edge weight happens to be equal (for example, if one phenotype is bimodal in a particular edge weight), ANOVA and Kruskal–Wallis will fail to identify this signal edge. To account for this deficiency, we advocate for the use of Distance Correlation (Dcorr)—a previously established universally consistent nonparametric test for equality in distribution (Panda et al., 2020;Székely et al., 2007)—to detect signal edges. Dcorrmeasures the strength of both linear and nonlinear association between two random variables (in this case, the edge weight and the phenotypic label). Furthermore, Dcorrequals zero if and only if the random variables are independent (Székely et al., 2007), unlike Pearson’s correlation coefficient. This allows Dcorrto serve as the test statistic for an independence test. Finally, recent statistical results have shown that distance- and kernel-based methods are equivalent, allowing any independence test to be reformulated as ak-sample test for equality in distribution (Shen & Vogelstein, 2020b).

##### 4.1.1.1 Simulation study

**Fig. 2.**
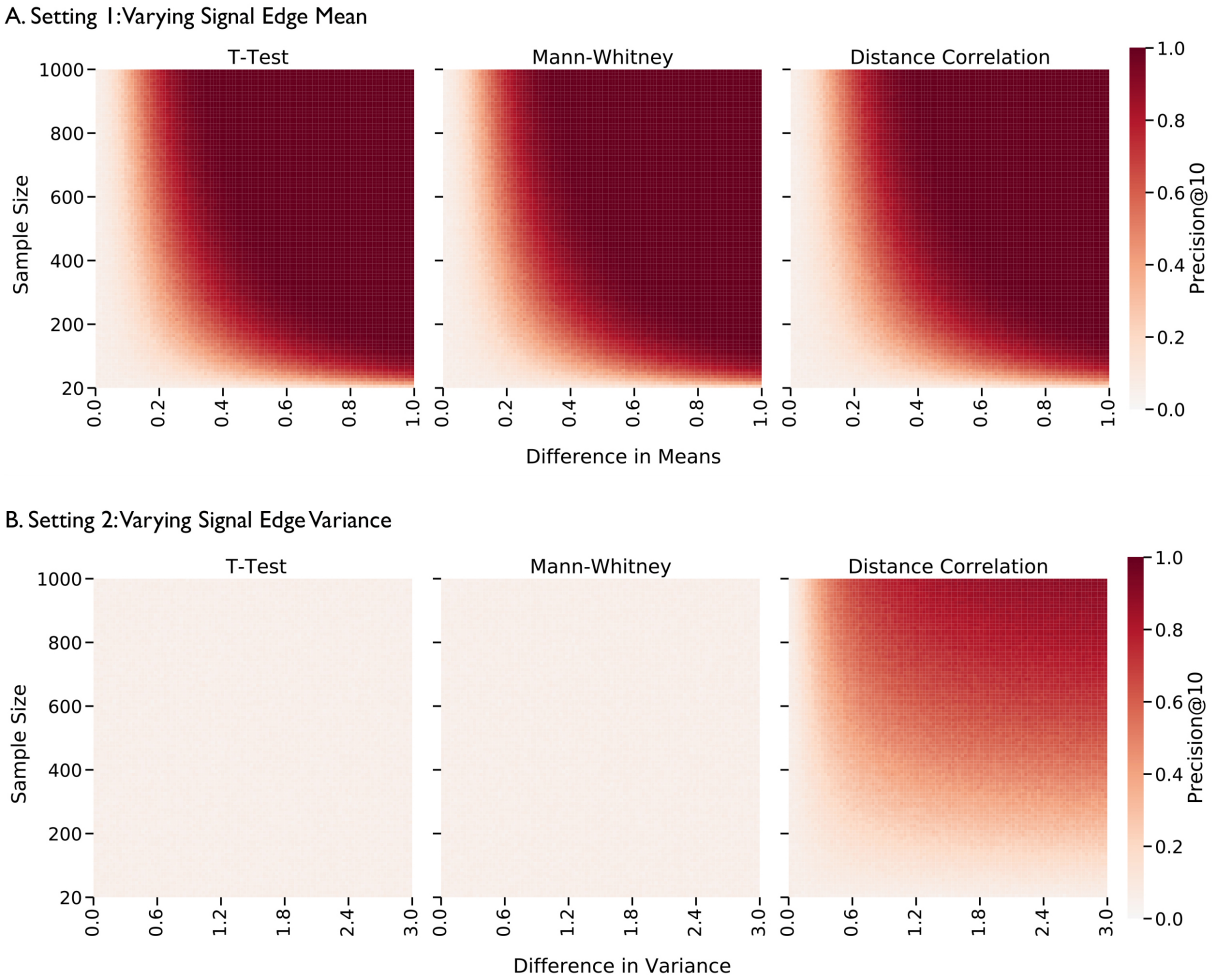
Precision@10 for each edge when comparing two populations of weighted networks using thet-test, Mann-Whitney U test, andk-sampleDcorrtest. The color bar represents precision averaged over 100 trials. (A) Results for varying the meanδand sample size while keeping the variance constant (ϕ=0). In this setting, all three tests perform similarly, and can detect signal edges when edge distributions differ in means. (B) Results for varying the varianceϕand sample size while keeping the mean constant (δ=0).t-testand Mann-Whitney test cannot detect changes in variance regardless of the sample and effect size.k-sampleDcorrtest is the only test that can detect signal edges with changes in variance.

To illustrate the advantages ofDcorr, we consider a two-population simulation setting where edge weights are sampled from distinct truncated normal distributions. When all signal edges are different only in their mean,Dcorr, ANOVA, and Kruskal–Wallis all successfully identify signal edges, and no test outperforms the others in this setting (Fig. 2A). However, when the mean edge weight is held equal but the variance is different across groups, onlyDcorrsuccessfully detects the signal edges (Fig. 2B). ANOVA and Kruskal–Wallis fail in this scenario because both test for differences in*location*(the mean and median, respectively) across groups, whereasDcorrtests for differences in*distribution*. This is of particular concern because many connectomics methods which aggregate serial edge-wise tests (e.g., the network-based statistic and its various extensions (Baggio et al., 2018;Yoo et al., 2017;Zalesky et al., 2010)) use ANOVA to detect signal edges, and this theoretical result demonstrates a scenario in which these methods produce a spurious result. For all implementation details, see the Supplement (§A.1).

##### 4.1.1.2 Real data experiments

**Fig. 3.**
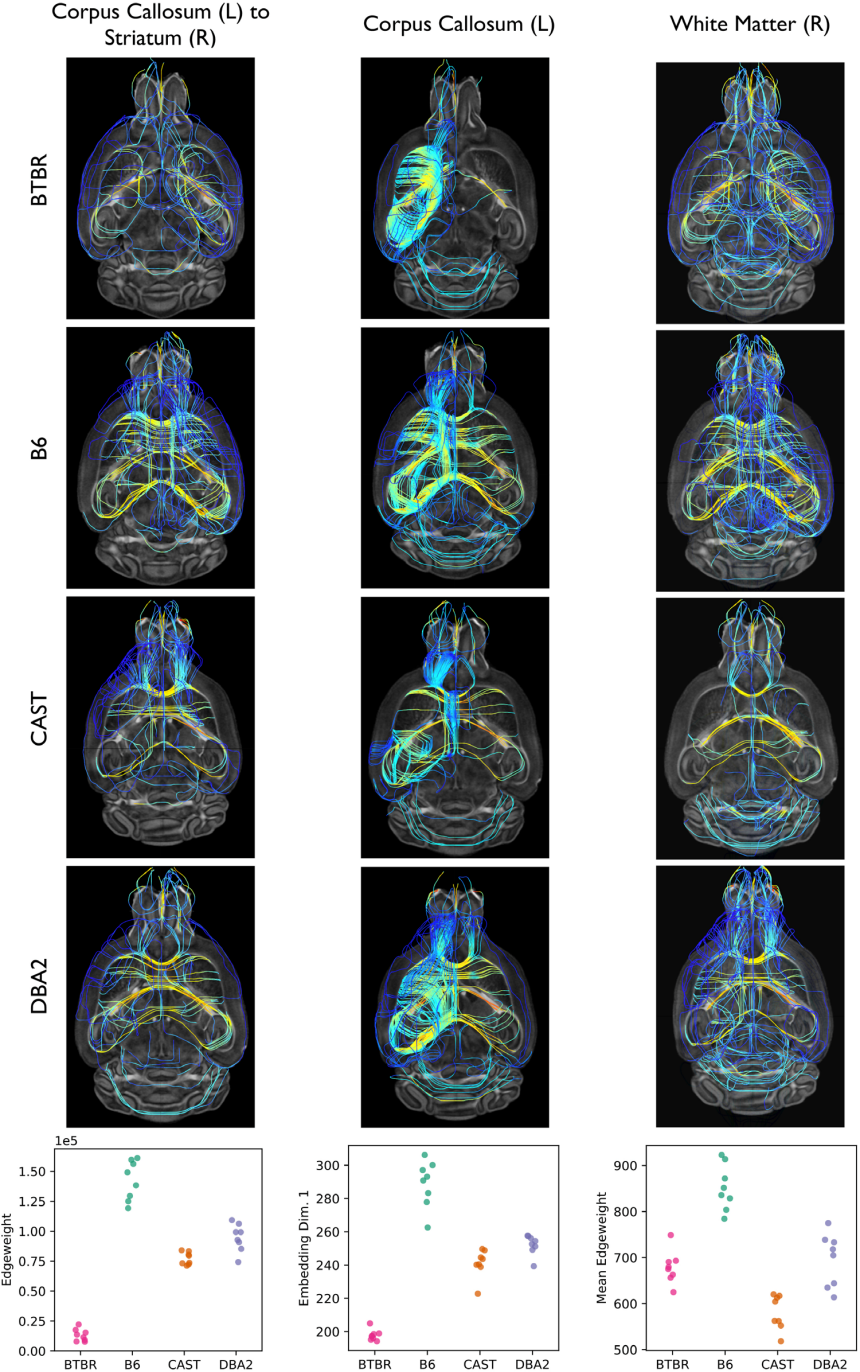
Visualization of the strongest signal edge (left hemisphere corpus callosum to right hemisphere striatum), vertex (left hemisphere corpus callosum), and community (right hemisphere white matter) across all mouse strains. At each topological scale, tractograms of these neurological structures are shown for each mouse strain. Bottom row: The distribution of edge weights for the strongest signal edge (Column 1); the distribution of the first embedding dimension for the strongest signal vertex (Column 2); and the distribution of average edge weight for the strongest signal community (Column 3). Each dot represents data from an individual mouse. Connective differences in the left hemisphere corpus callosum are apparent, with BTBR mice displaying a uniformly small vertex embedding. The average edge weight of the most significant community also shows pronounced variation across strains.

Using our edge-wise testing framework, we identified the signal edges across our four mouse strains. Application ofDcorrshowed that there was insufficient evidence to reject the null hypothesis of unequal edge weight distributions for any edge atα=0.05following Holm–Bonferroni correction. For more information on how we correct for multiple hypothesis testing, see the Methods (§3.2). However, this is more so a result of sample size and the stringency of family-wise error rate (FWER) control than it is a result of lack-of-signal: when we visualize the tractogram and edge weight distribution for the edge with the smallest p-value (left hemisphere corpus callosum to right hemisphere striatum), we can appreciate clear differences. For example, the edge is much sparser in the BTBR mouse and the spatial distribution of the edge in the mouse brain is highly heterogeneous across strain (Fig. 3). Therefore, to identify the*strongest*signal edges (i.e., the connections between brain regions whose wiring patterns were most heterogeneous across different genotypes), we use the ranking of the p-values for each edge. Note that choosing to rank either the p-values or the associated test statistics for a set of edges will produce the same outcome: the edges that are most connectively distinct across different groups will be top ranked in any of these metrics. For example, the edge with the smallest p-value (i.e., strongest signal edge) will also have the largest test statistic. Both of these metrics effectively act as pseudo-dissimilarity measures, quantifying the joint distance between an edge across groups.

A list of the 20 strongest signal edges, along with their correspondingDcorrtest statistics and p-values, are given inTable 1. The strongest signal edge connects the left hemisphere corpus callosum to the right hemisphere striatum. In fact, 11 of the 20 strongest signal edges are incident to these two ROIs, demonstrating that the connections emanating from these regions are highly heterogeneous across genotypes. Since connective abnormalities in edges are highly correlated with specific regions of the brain, this finding suggests that in addition to identifying signal*edges*, it is also insightful to interrogate the next hierarchical scale of network topology and identify signal*vertices*.

**Table 1.** The top 20 signal edges (out of 52,975 total edges) ranked by their Holm–Bonferroni corrected p-value.

Vertex 1	Vertex rank	Vertex 2	vertex rank	STATISTIC	p-value	Avg. vertex
Corpus callosum (L)	1	Striatum (R)	78	0.717	0.053	39.5
Corpus callosum (L)	1	Internal capsule (R)	8	0.699	0.070	4.5
Corpus callosum (L)	1	Reticular nucleus of thalamus (R)	70	0.698	0.072	35.5
Corpus callosum (L)	1	Zona incerta (R)	90	0.686	0.088	45.5
Septum (R)	32	Corpus callosum (R)	2	0.671	0.113	17.0
Striatum (L)	9	Striatum (R)	78	0.664	0.127	43.5
Corpus callosum (L)	1	Ventral thalamic nuclei (R)	35	0.663	0.128	18.0
Hippocampus (L)	40	Middle cerebellar peduncle (L)	128	0.658	0.139	84.0
Caudomedial entorhinal cortex (R)	73	Ventral hippocampal commissure (R)	82	0.656	0.145	77.5
Corpus callosum (L)	1	Midbrain reticular nucleus (R)	4	0.653	0.154	2.5
Midbrain reticular nucleus (L)	135	Superior cerebellar peduncle (L)	109	0.648	0.166	122.0
Corpus callosum (L)	1	Corpus callosum (R)	2	0.646	0.172	1.5
Spinal trigeminal Nerve (L)	81	Middle cerebellar peduncle (L)	128	0.645	0.173	104.5
Secondary visual cortex (L)	52	Striatum (R)	78	0.641	0.185	65.0
Globus pallidus (R)	25	Midbrain reticular nucleus (R)	4	0.632	0.217	14.5
Striatum (L)	9	Corpus callosum (R)	2	0.632	0.217	5.5
Primary somatosensory Cortex (L)	86	Secondary visual cortex (L)	260	0.629	0.228	173.0
Corpus callosum (L)	1	Primary somatosensory cortex (R)	178	0.628	0.230	89.5
Corpus callosum (L)	1	Ventral orbital cortex (R)	51	0.628	0.231	26.0
Zona incerta (R)	90	Intermediate reticular nucleus (R)	97	0.627	0.234	93.5

The ranks of the vertices incident to every signal edge are also annotated (seeTable 2for the strongest signal vertices). Eleven of the top 20 signal edges are adjacent to either the left or right hemisphere corpus callosum, the two strongest signal vertices.

#### 4.1.2 Identifying signal vertices

The ability to discover brain regions that are topologically dissimilar across phenotypes (i.e.,*signal vertices*) is critical for scientific and clinical analyses of connectomes, with broad applications such as the establishment of neurological biomarkers and identification of therapeutic targets (Craddock et al., 2015). Here, we leverage recent advances in the theory of random graph models to propose a principled and robust statistical method for identifying signal vertices. We also demonstrate that this method recovers more information about ROIs than vertex-level graph statistics, the predominant method for analyzing vertices in multi-subject connectomics (Craddock et al., 2013;Fornito et al., 2013;J. Wang et al., 2015). Specifically, we use the omnibus embedding (Omni) (Athreya et al., 2017)—a vertex embedding technique—to jointly represent all the connectomes in a multi-subject dataset within a common Euclidean subspace. This latent position quantifies a given vertex’s probability of connecting to any other vertex in the connectome. For more details, see the Methods (§2.2). For each vertex, we then apply a multivariate statistical test to determine whether the embedding of a specific brain region is different across groups. Previous results have shown that the latent position vectors estimated byOmniare asymptotically Gaussian (Levin et al., 2017), motivating our use of multivariate analysis of variance (MANOVA) to identify the signal vertices.

##### 4.1.2.1 Simulation study

To illustrate the advantages of this approach over existing techniques, we compareOmnito three other previously established vertex embedding methods: (i) the Exponential random graph model (ERGM), which incorporates the approach of representing each vertex with a vector of graph statistics (Simpson et al., 2011); (ii) Multivariate Distance Matrix Regression (MDMR), which represents each vertex as a vector of the weights for all adjacent edges (J. Kim et al., 2014); and (iii) the Network-Based Statistic (NBS), which identifies ROIs in subgraphs comprising exclusively strong signal edges as signal vertices (Zalesky et al., 2010). In a two-population simulation setting, we sample graphs from a distribution in which the true number of signal vertices is determined*a priori*. Then, for each vertex, we compute a p-value using each of the three vertex embeddings followed by Holm–Bonferroni correction. We measure the statistical power of each approach via a Receiver Operating Characteristic (ROC) curve, a performance metric which holistically describes the performance of a binary classifier by characterizing the trade-off between sensitivity and specificity as the discriminant threshold of the classifier (in this case,α) is varied. For all implementation details, see the Supplement (§A.2).

**Fig. 4.**
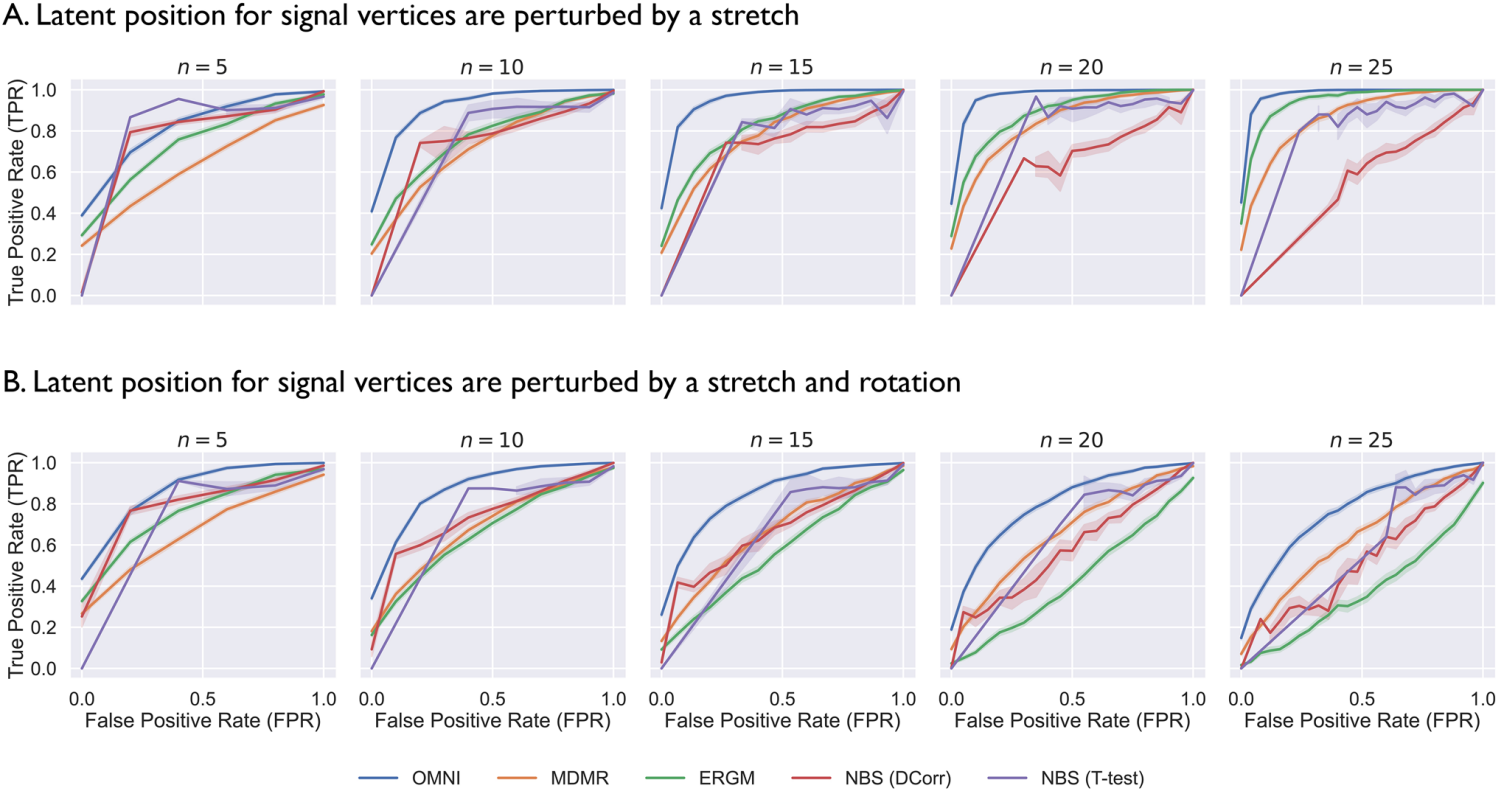
Ability of various vertex representations to identify signal vertices. The number of vertices in each network is kept constant (50), but the number of signal vertices is changed (n=5,10,15,20,25). For each vertex, we compute a p-value from each of the three vertex representations using Hotelling, and set the significance level atα=0.05following Holm–Bonferroni correction. Colors correspond to the method of vertex representation. (A) This setting compares two different RDPGs where the perturbed latent position is stretched by a constant. (B) This setting compares RDPGs where the perturbed latent position is rotated and stretched by a constant. Both settings show thatOmnisuccessfully differentiates null and signal vertices (AUC=87%) with much more accuracy than MDMR (AUC=73%), NBS (AUC=59% with thet-test), or the ERGM (AUC=68%). Replacing thet-testin NBS withDcorrmarginally improved the AUC to 62%, but the performance of this method is still far worse than thanOmni.

Figure 4shows thatOmniis a superior vertex representation method to the ERGM, MDMR, or NBS in this simulation, with an average Area Under the ROC (AUROC) 20% greater than the other three methods. Even in the most challenging simulation settings we test, where the true number of signal vertices is very small,Omnicorrectly identifies the vertices of interest with much higher accuracy than the other three methods. The ERGM, in particular, performed very poorly, achieving accuracy on par with random change in the most challenging settings (Fig. 4*bottom right panel*). We hypothesize that the poor performance of the ERGM results from the arbitrary choice of graph statistics used to parameterize the model. AsSimpson et al. (2011)note in their original ERGM paper, “the most appropriate explanatory metrics vary by network”. Thus, choosing an appropriate set of graph statistics for a given multi-subject connectomics dataset is a highly subjective task, limiting reproducibility of this method across studies. While a given graph statistic might be insightful given*a priori*knowledge about the data one is analyzing, they are not the most informative vertex embedding for any generic multi-subject connectomics dataset. In contrast, theOmni-based test we propose is a dataset-agnostic prescriptive statistical procedure that does not require hand tuning.

##### 4.1.2.2 Real data experiments

As it has been previously reported that the BTBR mouse has significant neuroanatomical abnormalities in the corpus callosum (Ellegood et al., 2013;Meyza & Blanchard, 2017), we expect our method to identify this brain region as a strong signal vertex. Our results corroborate this hypothesis: across all mouse strains, the left hemispheric portion of the corpus callosum is the strongest signal vertex, and its right hemispheric counterpart is the second strongest (Table 2). Note that because this atlas is symmetric, the portions of the corpus callosum in each hemisphere are parcellated as two distinct ROIs. In contrast, existing connectomics methods like MDMR fail to identify this salient neurobioligical information: as shown inSupplementary Table 1, MDMR ranks the left corpus callosum as the 34th strongest signal vertex despite its pronounced connective differences across strains. We also attempt to use vertex-level graph statistics (degree, clustering coefficient, betweenness centrality, closeness centrality, and number of triangles) to identify the connective abnormalities of the corpus callosum: however, the corpus callosum was the 92nd strongest signal vertex using this method. Like other existing embedding techniques not based on random graph models, MDMR and vertex-level graph statistics do not enjoy the statistical advantages ofOmni, including its interpretability and theoretical foundations.

**Table 2.** The top 20 signal vertices (out of 326 total vertices) ranked by the order of their Holm–Bonferroni corrected p-values.

Vertex	Pillai	F	p-value
Corpus callosum (L)	2.591	32.91	5.09e-25
Corpus callosum (R)	2.556	29.95	1.09e-23
Fimbria (L)	2.440	22.64	7.27e-20
Secondary motor cortex (L)	2.438	22.54	8.21e-20
Midbrain reticular nucleus (R)	2.430	22.16	1.38e-19
Substantia nigra (R)	2.305	17.25	2.20e-16
Internal capsule (R)	2.304	17.23	2.29e-16
Secondary motor cortex (R)	2.297	16.99	3.40e-16
Cerebral peduncle (R)	2.247	15.51	4.34e-15
Internal capsule (L)	2.238	15.27	6.71e-15
Striatum (L)	2.236	15.23	7.13e-15
Lateral ventricle (L)	2.218	14.74	1.74e-14
Stria terminalis (R)	2.202	14.35	3.59e-14
Cerebellar white matter (R)	2.199	14.28	4.08e-14
Optic tracts (L)	2.186	13.96	7.52e-14
Subthalamic nucleus (L)	2.178	13.78	1.05e-13
Hippocampus (R)	2.177	13.76	1.08e-13
Stria terminalis (L)	2.177	13.75	1.11e-13
Frontal association cortex (L)	2.170	13.60	1.47e-13
Rostral linear nucleus (R)	2.165	13.47	1.88e-13

Pillai’s trace and approximateFstatistic (along with 15,78 degrees of freedom) as calculated by one-way MANOVA are also reported. The left and right hemispheric components of the corpus callosum are the top two signal vertices, respectively.

InFigure 5, we plot the vertex embedding of the corpus callosum obtained byOmniusing a pairs plot (Emerson et al., 2013). BecauseOmniembeds each vertex of the graph ind-dimensional space, the pairs plot allows us to visualize the high-dimensional relationships in this Euclidean representation of network connectivity. Each dot represents the embedded corpus callosum of an individual mouse. These plots show scatter plot matrices on the off-diagonal panels, with kernel density estimates (KDEs) of the marginal distributions (smooth approximations of the underlying distribution of the data) on the diagonal. In the lower triangle of each pairs plot, we show 95% prediction intervals for each strain’s vertex embedding. Together with the KDEs, these figures show a high degree of separability in the embeddings, highlighting the intra-strain heterogeneity of the corpus callosum. Thus,Omnisuccessfully recovers a distinct representation of the corpus callosum in BTBR mice, and the corroboration of known neurobiological results adds further validation to this approach. For comparison, inSupplementary Figure 3, we also show pairs plots of two weak signal vertices: the left hemisphere cingulate cortex area 29c (rank 170 of 326) and the right hemisphere fasciculus retroflexus (rank 257 of 326). Mice from distinct genotypes are much less separable in embeddings of these vertices compared to the embeddings of the corpus callosum.

**Fig. 5.**
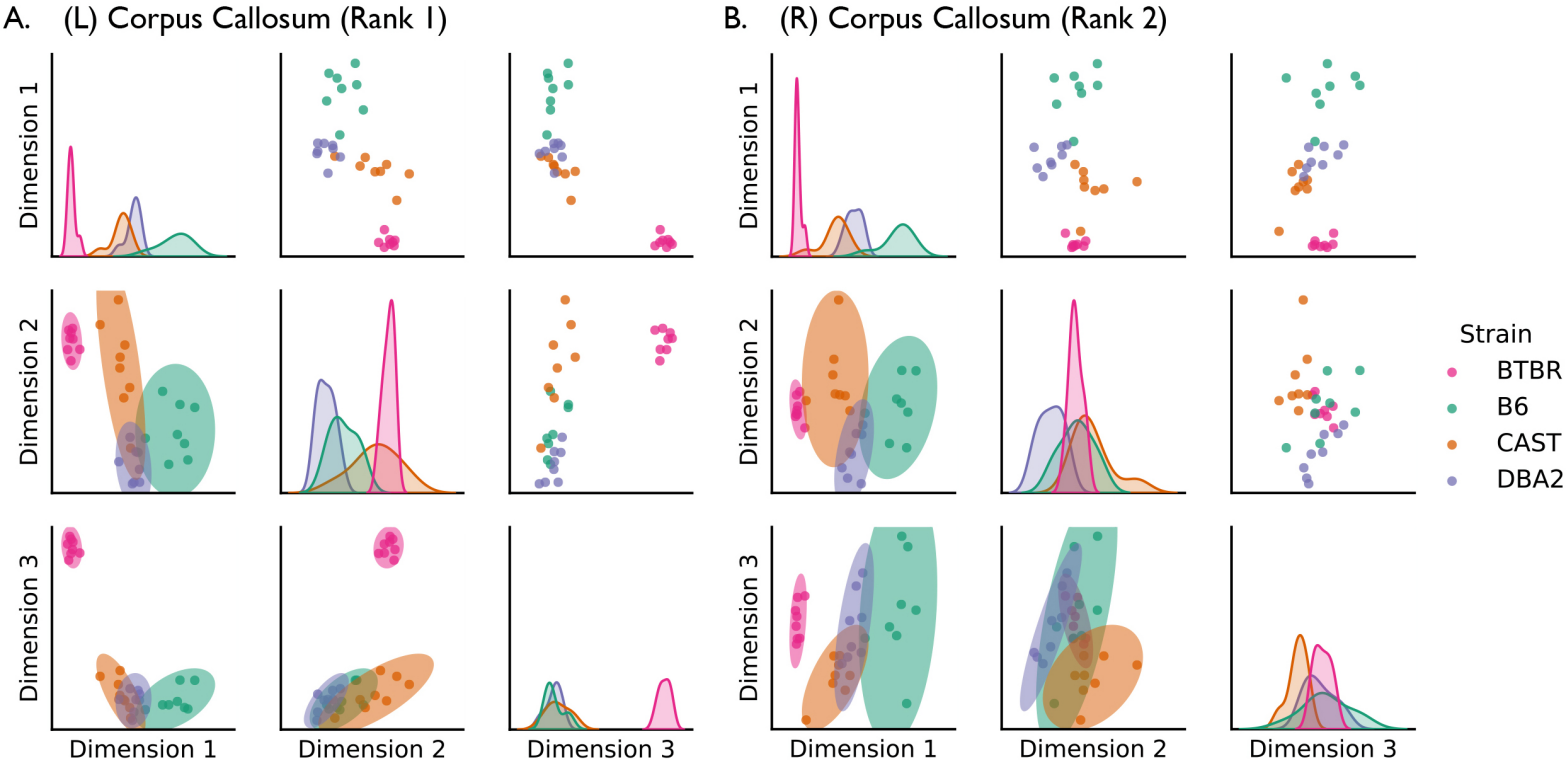
Pair plots of vertex embeddings of the (A) left and (B) right hemisphere corpus callosum produced byOmniacross all mouse strains. In BTBR, a behavioral model of autism, the corpus callosum has highly penetrant neuroanatomical defects. Compared to the wild-type models (B6, CAST, and DBA2), embeddings of BTBR’s left corpus callosum (the strongest signal vertex) are clearly differentiated. The same effect is present in the right corpus callosum, the second strongest signal vertex. Interestingly, corpus callosum embeddings from the wild-type mice are also separable (particularly the B6 strain), suggesting a diversity in corpus callosum architecture across the behavioral controls. Ellipses represent 95% prediction intervals for each strain.

Identification of the signal vertices also facilitates the discovery of interesting trends at finer resolutions of the connectome via multiscale analysis. For example, inTable 1, we observed that (unsurprisingly) many of the strongest signal edges are also incident to the corpus callosum, the strongest signal vertex. However, many of the most connectively distinct edges are incident to comparatively weak signal vertices. For example, the 9th strongest signal edge (from the right caudomedial entorhinal cortex to the right ventral hippocampal commissure) connects the 73rd and 82nd strongest signal vertices, respectively. In total, only 56% of the 100 strongest signal edges are incident to a top-20 signal vertex. This suggests that, while the average connectivity of a vertex may appear unremarkable, specific edges incident to that vertex may be pronouncedly correlated with phenotypes of interest.

Connectomes in this dataset are bilateral: for each ROI, there is a corresponding structure in both the left and right hemispheres. Therefore, we can aggregate the connective abnormality of a structure across hemispheres, enabling the identification of pairs of vertices that are significant in both the left and right hemisphere. InSupplementary Table 2, we provide a list of the 10 strongest signal vertex pairs, which we term bilateral signal vertices. For many bilateral signal vertices, the ROI in one hemisphere is often a much stronger signal vertex than the other hemisphere (for example, the right hemisphere cerebral peduncle is the 9th strongest signal vertex, while its right hemisphere counterpart is ranked 63rd).

### 4.2 Interrogating the *regional scale*

Communities of highly interconnected vertices are important structures within connectomes that underlie diverse neurological functions (Betzel et al., 2018;van den Heuvel et al., 2016). Therefore, communities are an important topological scale at which connectomes can be analyzed.

#### 4.2.1 Identifying signal communities

For analysis of connectomes where vertices are organized using an*a priori*community grouping, we compare four approaches for modeling the connectivity information encoded within a community; see the Methods (§3.1). Each successive approach yields a more holistic summary of the community. The first two approaches are univariate, comparing either (1) the probability of connectivity in a community or (2) the average edge weight in a community across populations. While these are fundamental properties of a community, summarizing the behavior of a community with a single scalar loses information. The last two approaches are multivariate, comparing either (3) the indices of nonzero edges in a community or (4) the vector of edge weights in a community. Note that the operations performed by approaches (1) and (3) require the connectomes to be binarized via Otsu’s method (Otsu, 1979), whereas approaches (2) and (4) operate on weighted connectomes. To test if the summarized information in a block is different across phenotypes, we again useDcorr.

##### 4.2.1.1 Simulation study

**Fig. 6.**
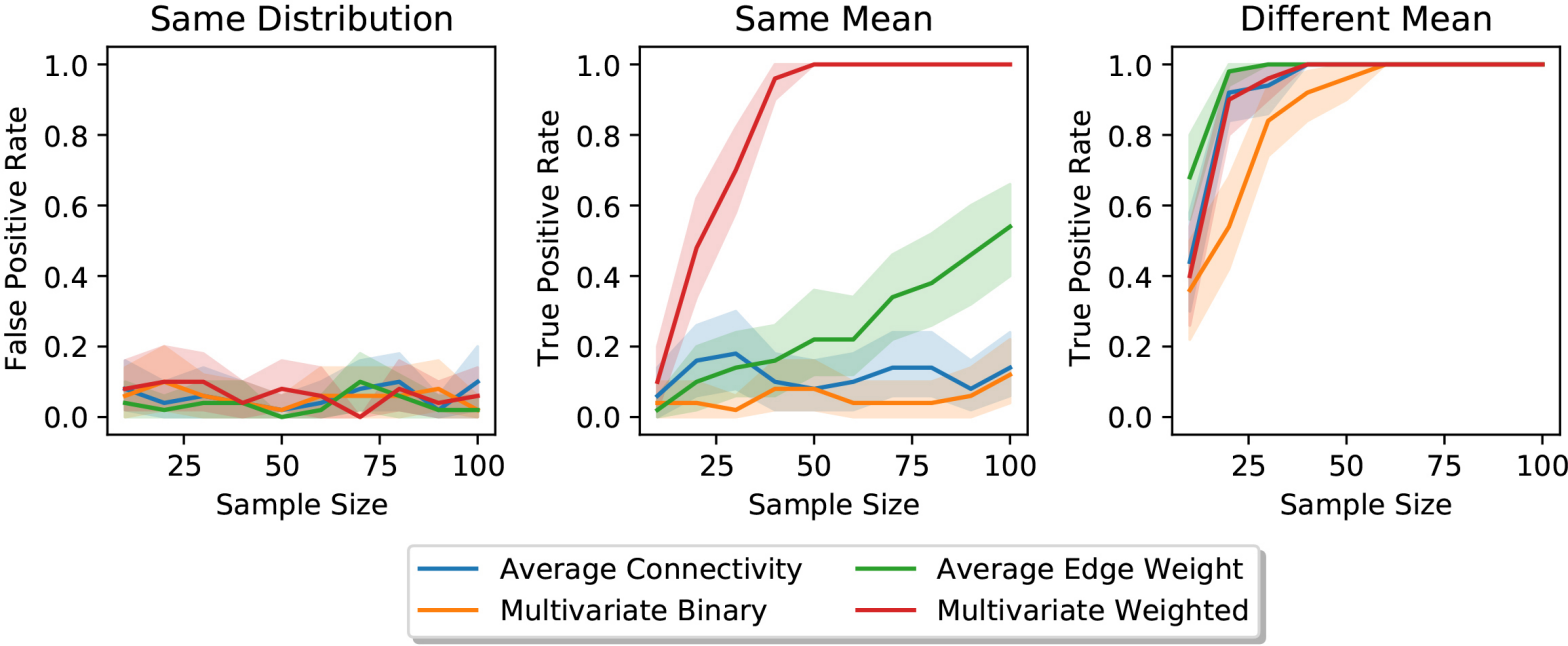
The Multivariate Weighted approach for signal community detection is superior to other proposed methods. We consider a two-population simulation where edge weights are sampled from 3-community block diagonal SBM. Distributions in the first block are equal, allowing us to measure the False Positive Rate (FPR) for each method. We see that all methods achieve a FPR less than or equal toα(left). In the second block, the two distributions have the same mean, but different variances. The Multivariate Weighted method is the most able to detect community edges in this setting; however, it requires a sample size of at leastN=25(middle). In the third block, the two distributions have different means, and in this setting, the Average Edge Weight method is superior given small sample sizes (N<25) (right). However, when the samples size is sufficiently large, the Multivariate Weighted method is equivalent. These results demonstrate the superiority of the Multivariate Weighted method over other proposed methods.

We compare these approaches in a two-population simulation setting; see the Supplement (§A.3). All methods were robust to false positives (Fig. 6, left); however, they differed in their ability to successfully identify signal communities. In settings where edge weights in a community have the same mean but different variances, univariate and binary approaches struggle to identify signal communities, achieving a maximum True Positive Rate (TPR) of 60%. However, comparing multivariate weighted representations of communities proved much more successful with a TPR of 80% for sample sizesN>30(Fig. 6, middle). When edge weights in a community have different means, all algorithms are able to successfully identify signal communities with a high TPR. However, only the weighted approaches can do this with small sample sizesN<25(Fig. 6, right). Therefore, we propose usingDcorrto compare communities across subjects in*regional scale*analyses.

##### 4.2.1.2 Real data experiments

InSupplementary Figure 2, we show log-transformedp-values obtained by the four approaches described above. Regions in blue are significant at the Holm–Bonferroni correction. Consistent with the simulations in the Supplement (§A.3), we see that univariate tests find less signal communities than the multivariate tests.p-values for all communities are given inTable 3. The strongest signal community (i.e., the one with the most heterogeneous topology across genotypes) determined byDcorris the intraconnection within the right hemisphere white matter. In fact, the majority of the 10 strongest signal communities involve connections to the white matter in both hemispheres.

**Table 3.** The top 10 signal communities (out of 105 total communities) ranked by the order of their Holm–Bonferroni corrected p-values as calculated by the multivariate weighted method.

Community 1	Community 2	Statistic	p-value
White matter (R)	White matter (R)	0.885	6.43e-06
White matter (L)	White matter (L)	0.857	1.02e-05
Hindbrain (L)	White matter (L)	0.849	1.14e-05
Midbrain (R)	White matter (R)	0.845	1.21e-05
Isocortex (L)	Isocortex (L)	0.844	1.22e-05
Pallium (R)	White matter (R)	0.831	1.50e-05
Isocortex (R)	White matter (R)	0.823	1.68e-05
Isocortex (R)	Isocortex (R)	0.819	1.80e-05
Isocortex (L)	White matter (L)	0.811	2.02e-05
Hindbrain (R)	White matter (R)	0.810	2.02e-05

### 4.3 Identifying multiscale differences in network architecture across genotypes

Using the statistical methods described above, we discover differences in brain connectivity between the four mouse genotypes at each topological scale of the connectome.Figure 3visualizes the strongest signal edge (as detected byDcorr), the strongest signal vertex (as detected byOmniand MANOVA), and the strongest signal community (as detected byDcorr) using tractograms, renderings of nerve tracts measured in the original DTI data. For an edge, the tractogram represents all the tracts between its two incident vertices; for a vertex, the tractogram represents all the tracts originating from that vertex; and for a community, the tractogram represents all the tracts interconnecting the vertices in a superstructure. All parameters used to generate these tractograms are available in the Methods (§3.4).

Tractograms allow us to visualize the heterogeneity in brain connectivity identified by our multiscale algorithms. For example, a tractogram of the left corpus callosum (Fig. 3, middle column) in the BTBR mice reveals a near absence of cross-hemispheric connections, while all control strains display much more cross-hemispheric connections at this vertex. In contrast,Supplementary Figure 4shows tractograms for the weakest signal edge, vertex, and community. In comparison to the tractograms shown inFigure 3, the tractograms of weak signal components are much more homogeneous across strains. Note, in the calculation of the weakest signal edge, we ignore edges with zero edge weight for all connectomes.

In addition to tractograms, we also plot the distribution of neurotopological features (edge weights and embeddings) used to determine the strongest signal structure at each scale (Fig. 3*,*bottom row). These distributions highlight the differences in connectome structure that each algorithm used to quantify the signal strength of that specific edge, vertex, or community.

### 4.4 Interrogating the *global scale*

**Fig. 7.**
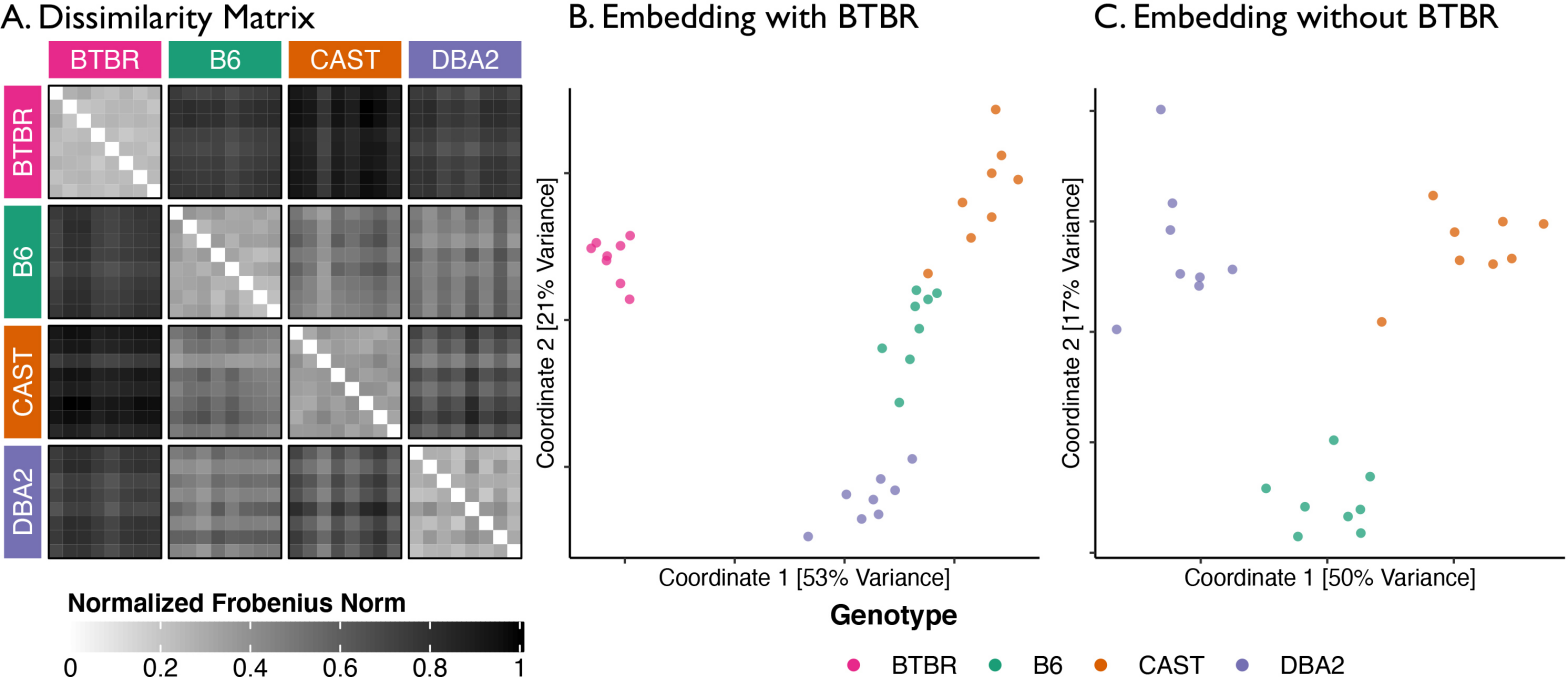
Pairwise dissimilarity between each mouse connectome, organized by mouse strain (A) and the joint embeddings of each sample with and without connectomes from BTBR mice in a two-dimensional space (B and C, respectively). Joint embeddings of every connectome were obtained using the omnibus embedding. (A) The pairwise dissimilarity between connectomes is calculated as the Frobenius norm of the difference between the embeddings of a pair of connectomes. (B) Two-dimensional representations of each connectome were obtained by using Classical Multidimensional Scaling (cMDS) to reduce the dimensionality of the embeddings obtained byOmni. (C) Same as center, but without data from BTBR mice.

To complete a multiscale analysis of multi-subject connectomics data, we demonstrate how results from our prior scales of network topology can be aggregated to enable comparisons of patterns in whole-brain connectivity across subjects.Figure 7Ashows the dissimilarity between each pair of connectomes in our dataset, where pairwise dissimilarity between connectomes$G_{1}$and$G_{2}$is calculated as the Frobenius norm of the difference between the corresponding embedding of each connectome calculated byOmni. Note that the dissimilarities inFigure 7Aare standardized by dividing by the largest pairwise dissimilarity. The average intra-strain dissimilarity (27%) is smaller than the average inter-strain dissimilarity (67%), confirming that connectomes from mice of the same genotype are globally most similar to one another. Additionally, the BTBR mice are very dissimilar to all other strains (the average inter-strain dissimilarity for the BTBR mice is 79%) while the three control strains are all fairly similar to each other (the average inter-strain dissimilarity for B6, CAST, and DBA2 mice is 55%).

We further reduce the dimensionality of these embeddings by using classical Multidimensional Scaling (cMDS) to embed this dissimilarity matrix into a two-dimensional space (Cox & Cox, 2008). This yields a collection of 32 points in${\mathbb{R}}^{2},$where each point represents the connectome of an individual mouse. The BTBR mice are highly separated from the other three control strains (Fig. 7B); all wild-type strains are also clearly distinct from each other (Fig. 7C). Thus, we can leverage information fromOmnito successfully differentiate all connectomes based on genotype, enabling comparisons of brain connectivity at the*global scale*.

### 4.5 The topology of vertices encodes information beyond the anatomy of those vertices

Finally, we demonstrate that the characterization of vertex-scale brain connectivity provided by the omnibus embedding contains topological information that is not available in commonly-used anatomical features. Following registration of all diffusion imaging data, the following anatomical features were derived for each vertex in every mouse brain: volume, apparent diffusion coefficient (ADC), fractional anisotropy (FA), and radial diffusivity (RD). For every vertex, we tested if the genotype labels and the low-dimensional embedding of the vertex produced byOmniwere conditionally independent given any of the anatomical features of that vertex (X. Wang et al., 2017).

Phrasing this mathematically, letY∈Ybe a discrete random variable representing the genotype of a mouse. For a given vertexi, let$X_{i}\in X\subseteq {\mathbb{R}}^{d}$be a random variable representing the vertex’s latent position in an RDPG estimated byOmni, and let$A_{i}\in A$be a vector of anatomical features that describe the vertex. If the mouse’s genotype and the vertex’s embedding are conditionally independent given the anatomical features, then

$$(Y1X_{i})|A_{i}\Rightarrow \text{Pr}(Y|X_{i},A_{i})=\text{Pr}(Y|A_{i}).$$ (3)

That is, the information about connectivity encoded in the vertex’s latent position is redundant given the anatomical features. However, if null hypothesis of conditional independence is rejected, the latent position contains information about connectivity not represented in the anatomy.

**Fig. 8.**
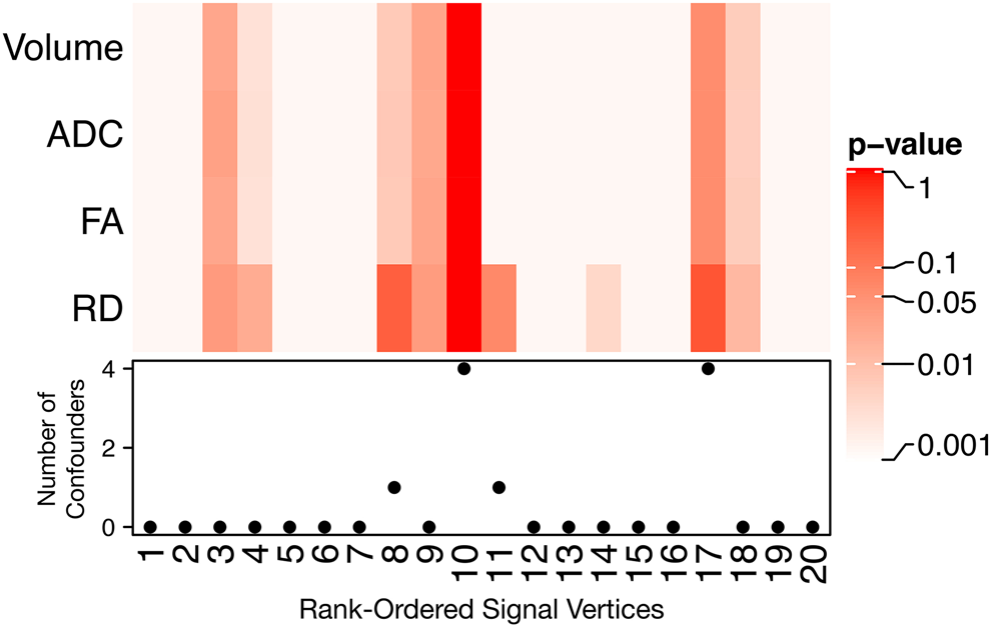
The omnibus embedding provides a novel understanding of brain connectivity at the scale of individual vertices. Using diffusion imaging, the following metrics were measured for each vertex: volume, apparent diffusion coefficient (ADC), fractional anisotropy (FA), and radial diffusivity (RD). To determine if a given feature provided more information than the embedding, these four anatomical features were compared to vertex embeddings produced byOmnivia a conditional independence test. The Holm–Bonferroni corrected p-values for conditional independence tests for each of the top 20 strongest signal vertices (Table 2) are shown on the heatmap above. A cell is red if genotype and the embedding are conditionally independent given the feature, implying thatOmniis confounded by the anatomical feature. The total number of confounding anatomical features for each vertex, sorted by signal strength, is shown below the heatmap.

We find that for 282 out of 326 vertices, the genotype labels andOmniembedding were conditionally dependent given each of the anatomical feature (Fig. 8). All four anatomical features were confounders for only 28 vertices, and the majority of these vertices were not in the top 100 strongest signal vertices. Our analysis also comports with previously described neuroanatomical changes in autism mouse models. For instance, the two top-20 signal vertices that are also confounded by ROI volume (left internal capsule and right hippocampus) have been shown to have marked volumetric changes in BTBR mice relative to wild-type strains (Ellegood et al., 2015). Thus, for most vertices, the omnibus embedding provides orthogonal insight into neural connectivity beyond what is encoded by the voxel-scale anatomical features, demonstrating the novelty of this method for*local scale*analysis.

## 5 Discussion

Robust and interpretable statistical methods for analyzing the network topology of connectomes are critically important to understand how patterns in brain connectivity are associated with observable neurological phenotypes. To this end, we leverage recent advances in the theory of random graph models to develop open-source methods that deliver statistically principled and interpretable analyses of multi-phenotype and multi-subject connectomics datasets. Specifically, these methods can be used to identify signal edges, vertices, and communities—that is, the components of the connectome across multiple topological scales that characterize the differences in network architecture observed between samples from distinct phenotypic backgrounds. Additionally, we have formulated these methods ask-sample hypothesis tests, meaning they can be used to jointly analyze connectomes from more than just two dimensional or categorical phenotypes.

There are numerous advantages to using random graph models to perform hypothesis tests on connectomes:

1. Hierarchical random graph models enable*multiscale modeling*of connectomes;
2. Random graph models are generative, meaning the estimated model parameters characterize the structure of a particular scale of network topology, and are therefore*interpretable*;
3. Unlike many other connectomics methods, random graph models have*provable properties*which motivate robust and principled downstream statistical analyses.

For example, consider the identification of signal vertices using the Random Dot Product Graph (RDPG) andOmni. A previously demonstrated central limit theorem proved that the embeddings estimated byOmniare asymptotically normal (Levin et al., 2017), motivating our use of MANOVA to test for connective differences among the ROIs in a connectomics dataset. In contrast, vertex-level graph statistics have no such governing distribution. In fact, multiple studies have shown that connectomes with wildly different topologies can produce identical graph statistics (Chen et al., 2019;Chung et al., 2021)—à la Anscombe’s quartet (Anscombe, 1973)—so while graph statistics can produce seemingly intuitive and logical summaries of connectome connectivity, these measures are generally unable to provide a holistic characterization of a vertex’s connectivity. This is reflected in our simulation studies: successfully detecting signal vertices using vertex-level graph statistics is an under-powered statistical approach, with an average area under the ROC curve (AUROC) of 68% (Supplementary Fig. 4), lagging behind our proposed method, which achieves an average AUROC of 87%.

We have previously proposed vertex embedding methods other thanOmni, and while these methods have certain advantages overOmniin particular scenarios, they too lack many desirable statistical properties. For example, unlikeOmni, the Joint Spectral Embedding (Nenning et al., 2020) can embed connectomes with different numbers of vertices. While this is useful for cross-species comparisons (Xu et al., 2020), this paradigm is not often encountered in multi-subject connectomics datasets of the same species (e.g., mice or humans) given that the same atlas can be applied to all subjects. One disadvantage of the Joint Spectral Embedding, however, is that its outputs are not interpretable. InOmni, the vector embedding of a given vertex quantifies its probability of connecting to any other vertex in the connectome. In addition to lacking such interpretability, the Joint Spectral Embedding does not enjoy the asymptotic normality ofOmni, limiting its application for identifying signal vertices.

Our methods for identifying signal edges, vertices, and communities enable powerful multiscale analyses of connectomes. Additionally, we show how information from each of these scales can be aggregated to perform whole-brain comparisons of global connectivity (Fig. 7). Furthermore, our methods successfully recover previously known neurobiological information. In addition to identifying connective differences in BTBR, such as the aberrant corpus callosum (Fig. 3) and volume-associated changes in the internal capsule and hippocampus (Fig. 8), molecular mechanisms for connective differences in the substantia nigra (rank 6 of 326) have also been identified. Specifically, levels of anti-brain antibodies were elevated in the substantia nigra in BTBR compared to controls, implicating a potential neuroinflammatory axis for neuroconnective changes (S.-N. Kim et al., 2016). However, to the best of our knowledge, many of the connective differences identified by our methods do not have known neurobiological mechanisms. This demonstrates that our methods can be used to generate novel hypotheses from computational datasets for further biological investigation.

Previous methods have attempted to perform similar multiscale analyses, but, in addition to analyzing fewer scales than our methods are capable of, the theoretical foundations of these methods could be improved. For example, the network based statistic (NBS) aggregates the results from serial edgewise tests to find clusters of inter-connected vertices that are connectively different between two groups (Zalesky et al., 2010). However, NBS uses thet-testand arbitrary thresholding to determine which edges in the connectome are signal edges. As we have shown in our simulations, thet-test(as well as other tests of location, such as Kruskal–Wallis) can only successfully detect signal edges in well-behaved edge weight distributions (e.g., two normal distributions with equal variance and different means) (Supplementary Fig. 2). Edge weights can be distributed differently across phenotypes, and therefore be true signal edges, but have the same mean (or median), and NBS will fail to successfully identify them as signal edges. To correct for this deficiency, we advocate for the use ofDcorr, a nonparametric, universally consistent test for differences in distribution (Panda et al., 2020;Székely et al., 2007). By treating each edge weight as an independent random variable under the Independent Edge (IE) random graph model, we enable far more powerful edgewise testing. In the Supplement (§A.2), we substitute thet-testin NBS withDcorr. While this modification improves the method’s average AUC by about 3%, both versions of NBS fail to robustly identify signal edges, and lag behind existing methods like MDMR and our Omni-based procedure. If one is truly interested in estimating vertex clusters like those produced by NBS, we instead recommend the signal subgraph estimator (Vogelstein et al., 2013;S. Wang et al., 2018), which has the added benefits of being provably consistent, robust, and interpretable. Open-source Python implementations of the signal subgraph estimator, along with all of our methods, are freely available online (Chung et al., 2019).

Collectively, the proposed methods presented in this work motivate a number of potential future extensions. First, there is room to develop statistical innovations that will be able to determine if a particular random graph model appropriately models a given real-world dataset. This can be accomplished via tests of goodness-of-fit (GoF), an important criterion for model selection (Myung, 2000). A GoF test has already been developed for the inhomogeneous Erdős–Rényi random graph (Dan & Bhattacharya, 2020), a particular version of the IE model, and similar tests can be developed for the RDPG (vertex-scale model) and the SBM (community-scale model). Once developed, these tests can be used as a validation step before running any of our proposed multiscale tests, adding further rigor to these methods.

Second, whileDcorrenables statistically powerful edge- and community-wise testing, it is very computationally expensive. Performingk-sample testing withDcorrtypically requires a costly permutation test to estimate the null distribution and subsequent p-value. While there is an accurate chi-square approximation for the null distribution of unbiasedDcorrwith comparable finite-sample power (Shen & Vogelstein, 2020a), this test generally requires a sample size≥20to be statistically valid. Therefore, in addition to rapidly gaining power with additional samples, our methods also enjoy boosts to computational efficiency at sufficiently high sample sizes. However, in connectomics, it can sometimes be difficult to achieve a sample this large, particularly if one is studying a rare neurological disorder or using a very time-intensive process to estimate the connectome.

Third, these methods are designed for the comparison of samples of connectomes from distinct categorical or dimensional phenotypes. Statistical modeling of the connectome in relation to a continuous phenotypic variable of interest (such as age) is a fundamental challenge for the analysis of dynamic connectomes. While our methods can be applied if the continuous variable is discretized into categorical bins, extensions are required to enable true regression analysis of the connectome in this statistical modeling framework.

## 6 Conclusion

The network-level view of brain organization provided by the connectome will enable a transformative understanding of the brain (Morgan & Lichtman, 2013). For an organ system whose function—both in disease and in health—remains so poorly understood, the promise of this new data type is immense. However, our ability to*map*connectomes is quickly outpacing our ability to*analyze*them. As the wealth of neuroimaging and connectomics data continues to grow, new mathematical and statistical techniques will be required to discover the brain circuits that underlie neurological processes, disorders, and diseases. We anticipate that the multiscale algorithms and techniques presented in this work will be widely used by future researchers to uncover the neurobiological correlates of different phenotypes in multi-subject connectomics datasets.

## Supplementary Material

Supplementary Material

## Data Availability

The multi-subject connectomics dataset analyzed in this paper was derived by Wang et al., and is described in further detail in the original publication (N. Wang et al., 2020). These data are freely available in graspologic (https://github.com/microsoft/graspologic) (Chung et al., 2019), an open-source Python package for statistical network analysis. All network-related analyses and simulations were performed using graspologic, and all multivariate hypothesis testing was performed using hyppo (https://github.com/neurodata/hyppo) (Panda et al., 2020). The code necessary to reproduce the analyses, simulations, and figures presented in this work is available in a series of Jupyter Notebooks athttps://github.com/neurodata/MCC.
