# Supplementary material for: Multiscale comparative connectomics
Source: Imaging Neurosci (Camb). 2025 May 16;3:IMAG.a.2. doi: 10.1162/IMAG.a.2 (PMC12319836; doi:10.1162/IMAG.a.2)
Supplement: Supplementary Material [file imag.a.2_supp.pdf]

Multiscale Comparative Connectomics (*Supplement*)

Vivek Gopalakrishnan[†1], Jaewon Chung[1], Eric Bridgeford[2], Benjamin D. Pedigo[1], Jesús Arroyo[3], Lucy Upchurch[4], G. Allan Johnson[4,5], Nian Wang[6], Youngser Park[7], Carey E. Priebe[7,8], and Joshua T. Vogelstein[1,2,7,8]

[1]Department of Biomedical Engineering, Johns Hopkins University
[2]Department of Biostatistics, Johns Hopkins University
[3]Department of Statistics, Texas A&M University
[4]Center for In Vivo Microscopy, Department of Radiology, Duke University
[5]Department of Biomedical Engineering, Duke University
[6]Department of Radiology and Imaging Sciences, Indiana University School of Medicine
[7]Center for Imaging Science, Johns Hopkins University
[8]Department of Applied Mathematics and Statistics, Johns Hopkins University
[†]*Correspondence to: vivekg@mit.edu*

A Simulations

A.1 Edge simulations. We consider two populations of networks generated from a two-community SBM. Edge weights are sampled from a truncated normal distribution to emulate correlation matrices. All networks have $n = 20$ vertices with 10 vertices belonging to the first community and 10 vertices belonging to the second community. The community probability matrix for each population is given by

$$\mathbf{B}^1 = \begin{bmatrix} \mathcal{TN}(0, 0.25) & \mathcal{TN}(0, 0.25) \\ \mathcal{TN}(0, 0.25) & \mathcal{TN}(0, 0.25) \end{bmatrix}$$

$$\mathbf{B}^2 = \begin{bmatrix} \mathcal{TN}(0+\delta, 0.25+\phi) & \mathcal{TN}(0, 0.25) \\ \mathcal{TN}(0, 0.25) & \mathcal{TN}(0, 0.25) \end{bmatrix}$$

where $\mathcal{TN}(\mu, \sigma^2)$ denotes a truncated normal distribution with mean μ and variance σ^2 such that all values are bounded within $[-1, 1]$. A total of $m = 20, 40, \ldots, 1000$ networks are sampled ($m/2$ networks per population). In the first population, all edges are sampled from the same edge weight distribution $\mathcal{TN}(0, 0.25)$. In the second population, all edge weights are also sampled from $\mathcal{TN}(0, 0.25)$ except for those in the first community. In this community, the distribution of edge weights has either a different mean, δ, or variance, $0.25 + \phi$, from the first population. Therefore, the edges in the first community of these simulated connectomes are the signal edges that we hope to correctly identify. For each edge, p-values are computed by three different tests: 1) T-TEST, 2) Mann-Whitney (MW) U test, a non-parametric test of medians, and 3) 2-sample Distance Correlation (DCORR) test, a test of equality in distribution. For each test, the p-values are sorted to find the ten most significant edges, and the performance is evaluated with precision (the number of signal edges that are correctly identified).

Figure 2 shows the Precision@10 as a function of sample size, mean, and variance. Figure 2 top row shows that all three tests can identify signal edges that are different in mean, and that no particular test is superior than another in this setting. Figure 2 bottom row shows that only DCORR can detect signal edges with differences in variance when the means are kept the same. This is because T-TEST and MW test for differences in location (e.g., mean or median), whereas DCORR tests for any differences between a pair of observed distributions. Because DCORR achieves about the same precision and recall as the location tests when only location varies, and demonstrates considerably better operating characteristics when the variance varies, we use it in the real-world data. Note that when looking at the top-k edges, the choice of k is arbitrary. The salient point from this simulation is that if we look at the top-10 edges identified by each algorithm, none of those edges selected by the T-TEST and MW are signal edges — on average, they are false positives.

If the sample size $m \geq 20$, one can use a chi-square test that well approximates the k-sample DCORR test Shen and Vogelstein (2020a). This can improve the computational efficiency of edge-wise testing by avoiding the costly permutation test that DCORR normally uses to estimate the null distribution.

A.2 Vertex simulations. One goal of connectomics is to identify signal vertices that are different between populations. In this section, we identify signal vertices using different vector-based vertex representations. The first representation we consider is the simplest: it is possible describe a vertex using all its incident edges via the corresponding row (or column) of a vertex in the adjacency matrices, and compare these vectors using Multivariate Distance Matrix Regression (MDMR) J. Kim, Wozniak, Mueller, Shen, and Pan (2014). The second representation we consider, which is highly popular in modern connectomics literature, is a set of vertex-level graph statistics. Specifically, we fit an exponential random graph model (ERGM) Simpson, Hayasaka, and Laurienti (2011) with the following graph statistics for each vertex: local clustering coefficient (LCC), betweenness centrality (BC), closeness centrality (CC), and number of triangles. The third representation we consider is the network-based statistic (NBS), which identifies signal vertices as those that comprise subgraphs of strong signal edges Zalesky, Fornito, and Bullmore (2010). In the traditional setup for NBS, signal edges are identified using a T-TEST (or ANOVA in multifactorial designs). Based on the results of our edge-wise simulation (§A.1), we also test the performance of NBS when the T-TEST is substituted for DCORR. The final representation we consider are the low-dimensional latent-space positions estimated by OMNI. Since all vertex representations are multivariate, hypotheses are tested using HOTELLING, a multivariate generalization of the T-TEST.

We consider a population of RDPG s in two different settings where the number of signal vertices is varied. In both

settings, m null vertices are sampled from the latent position $X_1 = [0.25, 0.25]$ and n signal vertices are sampled from $X_2 = \mathrm{rot}(70°)X_1$, where $\mathrm{rot}(\theta)$ represents a 2-dimensional rotation matrix of angle θ. In setting 1, X_2 is stretched, and a second RDPG is constructed with latent positions X_1 and $X_3 = 0.4X_2$ and an equivalent number of vertices per each position. In setting 2, X_2 is stretched and rotated (i.e., $X_3 = 0.4\mathrm{rot}(10°)X_2$). The vertices sampled from X_2 or X_3 (i.e., from different latent positions) are considered signal vertices, and we vary the number of these vertices from $n = 0, 5, 10, 15, 20, 25$. The number of null vertices is set to $m = 50 - n$. A total of 100 networks are sampled per population, and the p-values are computed using HOTELLING on each of the three vertex representations for each vertex. Vertices with p-values less than $\alpha = 0.05$ after Holm–Bonferroni correction are classified as signal vertices in this simulation. The performance of each algorithm is measured using a Receiver Operator Characteristic (ROC) curve, which shows the trade-off between the False Positive Rate and True Positive Rate for each method.

Figure 4 shows that OMNI is uniformly a more accurate method for identifying signal vertices. Across all settings, its accuracy (measured by the Area Under the ROC curve) is higher than the other three methods. In particular, the ERGM, which uses the widely popular approach of graph statistics, performs much worse at this task. Substitute the T-TEST in NBS with DCORR improves the method's ability to detect signal edges by about 3%, but the performance of both variants lags far behind OMNI.

A.3 Community simulations. We consider two populations of networks generated from a three-community block diagonal SBM. As in the edge simulation (Methods §A.1), edge weights are sampled from truncated normal distributions to emulate correlation matrices. All communities have $n = 10$ vertices each, and the community probability matrix for each population is given by

$$\mathbf{B}^1 = \begin{bmatrix} \mathcal{TN}(0, 0.25) & & \\ & \mathcal{TN}(0, 0.25) & \\ & & \mathcal{TN}(-0.75, 0.25) \end{bmatrix},$$

and

$$\mathbf{B}^2 = \begin{bmatrix} \mathcal{TN}(0, 0.25) & & \\ & \mathcal{TN}(0, 0.50) & \\ & & \mathcal{TN}(0.75, 0.25) \end{bmatrix}.$$

A total of $m = 10, 20, \ldots, 100$ networks are sampled ($m/2$ networks per population). Note that the edge weight distributions in the first diagonal block are equal, while the distributions have different variances in the second diagonal block and different means in the third diagonal block. Therefore, data from block one allow us to measure the false positive rate (FPR), while data from blocks two and three allow us to measure the true positive rate (TPR). Each simulation run was conducted 50 times to ensure accurate estimation of the FPR and TPR. All p-values were corrected using Holm–Bonferroni with significance determined at $\alpha = 0.05$.

From these simulations, we see that all four tests have roughly the same FPR (about 5% regardless of sample size) (Figure 6 *first panel*). However, they differ in their ability to detect signal communities. When all populations have the same mean edge weight, but different variances, only the Multivariate Weighted method consistently detects signal communities once the sample size is greater than 25 networks (Figure 6 *second panel*). All other methods have a lower TPR in this setting. When the edge weights have different means, the TPR of the Multivariate Weighted method lags behind the Average Edge Weight method in low sample size settings ($N < 25$) (Figure 6 *third panel*). However, when the sample size is larger than $N > 25$, the Multivariate Weighted and Average Edge Weight methods perform comparably. These simulations demonstrate that the Multivariate Weighted signal community detection method, which includes the most information about connectivity within a block, achieves the highest TPR while maintaining a FPR less than or equal to the significance value of the test.

B Supplementary investigations of real data

Visualizations of connectomes. The average adjacency matrix of each strain was computed and visualized as a heatmap (Supplementary Figure 1). Connectomes in this dataset are undirected and parcellated with a bilateral atlas.

Comparison of mesoscale methods. In Supplementary Figure 2, we show the log-transformed p-value for each approach, with communities in blue denoting signal communities (significant after Holm–Bonferroni correction).

Embeddings of weak signal vertices. The strongest signal vertex identified by this method in our real-world data was the left hemisphere corpus callosum. In Figure 5, we demonstrate how a *pairs plot*, a d-dimensional scatter plot matrix, can be used to visualize the vertex embedding produced by OMNI. In Supplementary Figure 3, we show pairs plots of two weak signal vertices for comparison.

Visualizing null edges, vertices, and communities.x Using the procedures described above, we identified the weakest signal edge (the left frontal cortex to the right temporal association cortex), vertex (the left medial longitudinal fasciculus and tectospinal tract), and community (left isocortex to left diencephalon) across all mouse strains. To contrast with the strongest signal structures shown in Figure 3, we plotted tractograms of the weakest components neurological structures (Supplementary Figure 4). These tractograms are much more homogeneous than those plotted in Figure 3, as are the distributions of numerical features for each component (shown in the bottom row). Since this edge, vertex, and community contain no information about the strain of a mouse, we term them *null components*.

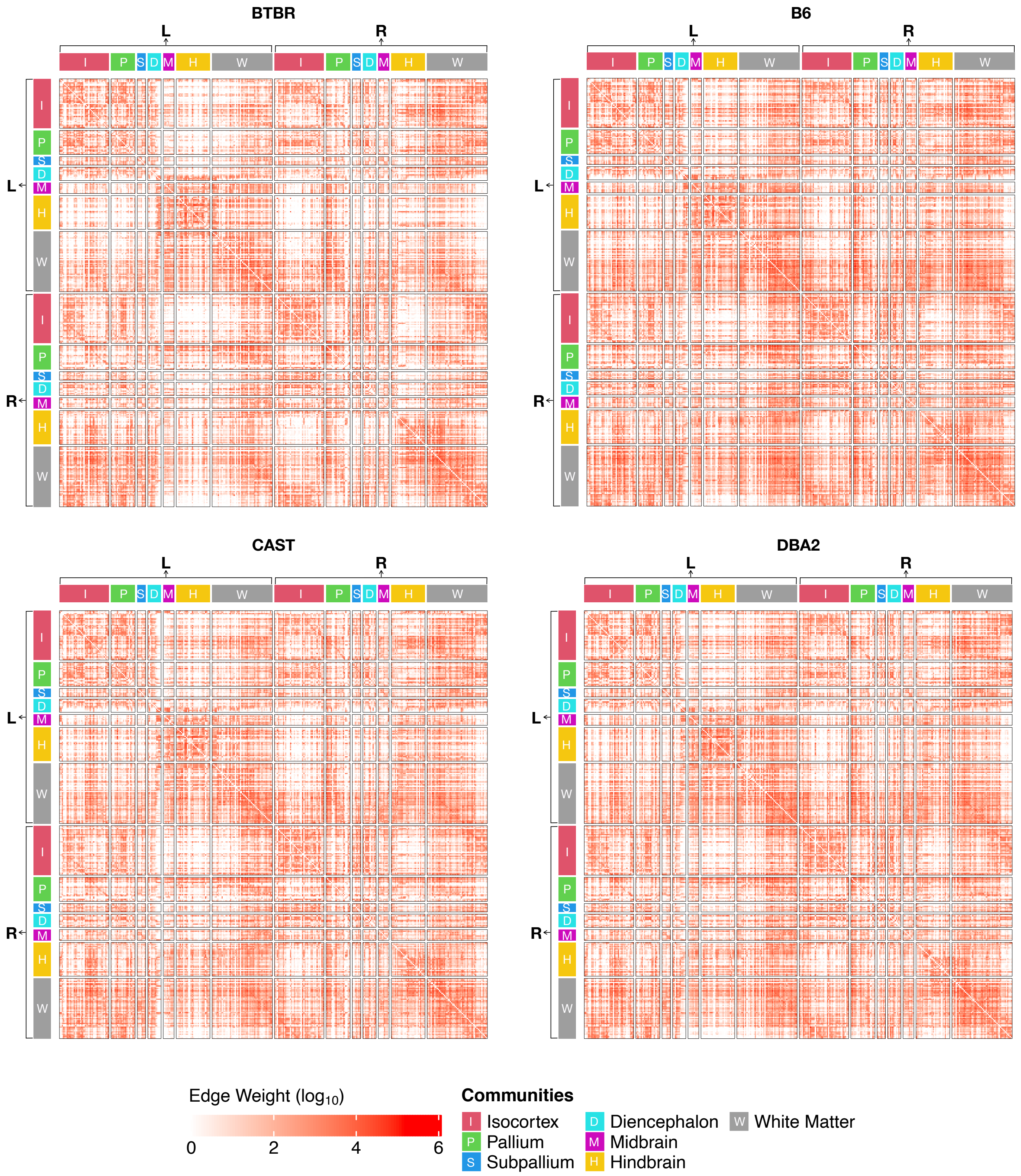

Supplementary Figure 1: Log-transformed average adjacency matrices for each mouse strain with hierarchical structure labels. Hierarchical labels show the hemispheric and superstructure level. For hemispheric labels, L (R) denotes the left (right) hemisphere. Superstructures are labeled as follows: I) isocortex, P) pallium, S) subpallium, D) diencephalon, M) midbrain, H) hindbrain, and W) white matter.

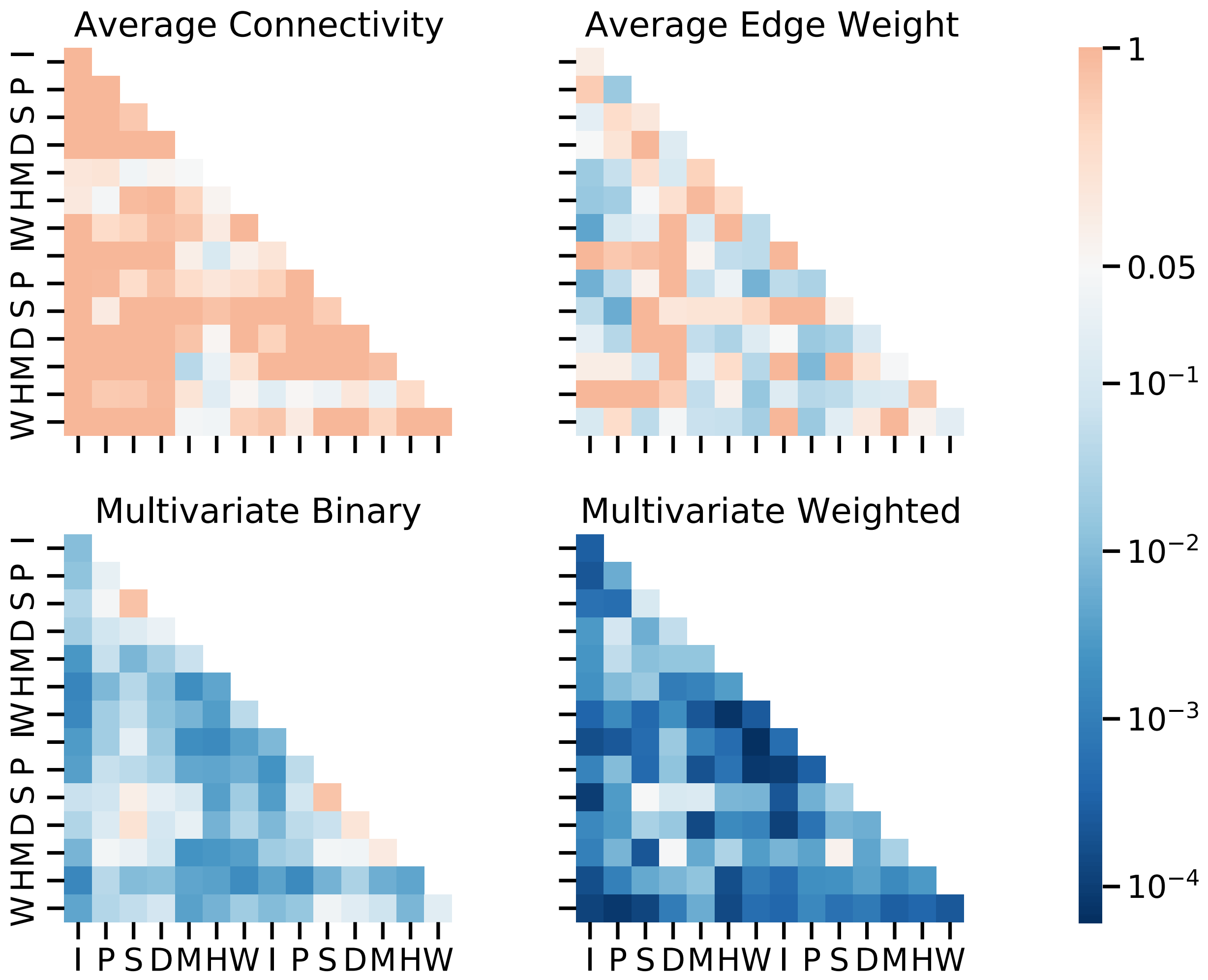

Supplementary Figure 2: P-values for each proposed approach for summarizing the information in a communities. More signal communities are found as the amount of information encoded by the summary method increases. Because connectomes in this dataset are undirected, this significance matrix will be symmetric and therefore only the upper triangle of these matrices should be considered. The colorbar represents $\log_{10} p$-values. Significance is determined at $\alpha = 0.05$ following Holm–Bonferroni correction; therefore, communities in blue are signal communities.

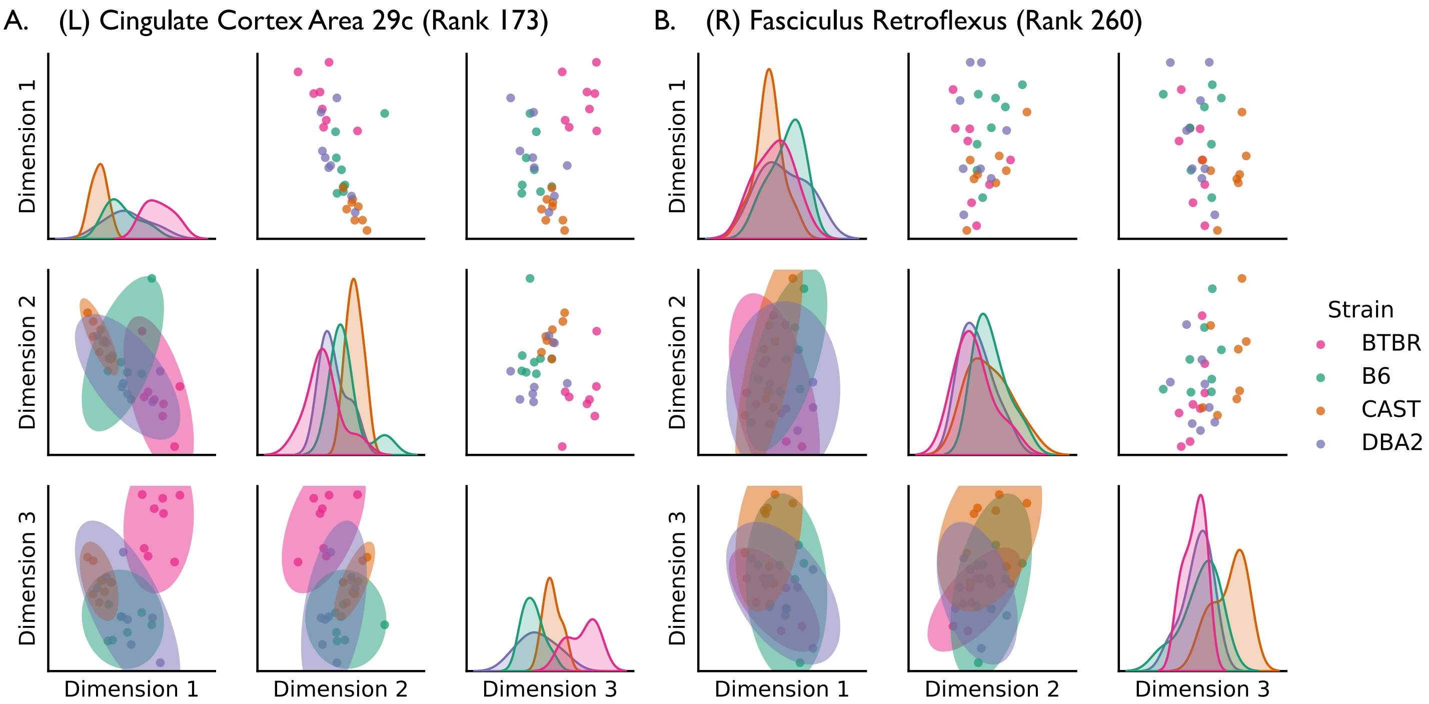

Supplementary Figure 3: Pairs plots of the vertex embeddings of the left cingulate cortex area 29c and the right fasciculus retroflexus, two weak signal vertices identified by `Omni` and `MANOVA`. As shown by the kernel density estimates (*diagonal*) and the 95% prediction ellipses (*lower triangle*), the distribution of these embeddings is not separable. This emphasizes the homogeneity of these vertices across strains relative to the two strongest signal vertices shown in Figure 5.

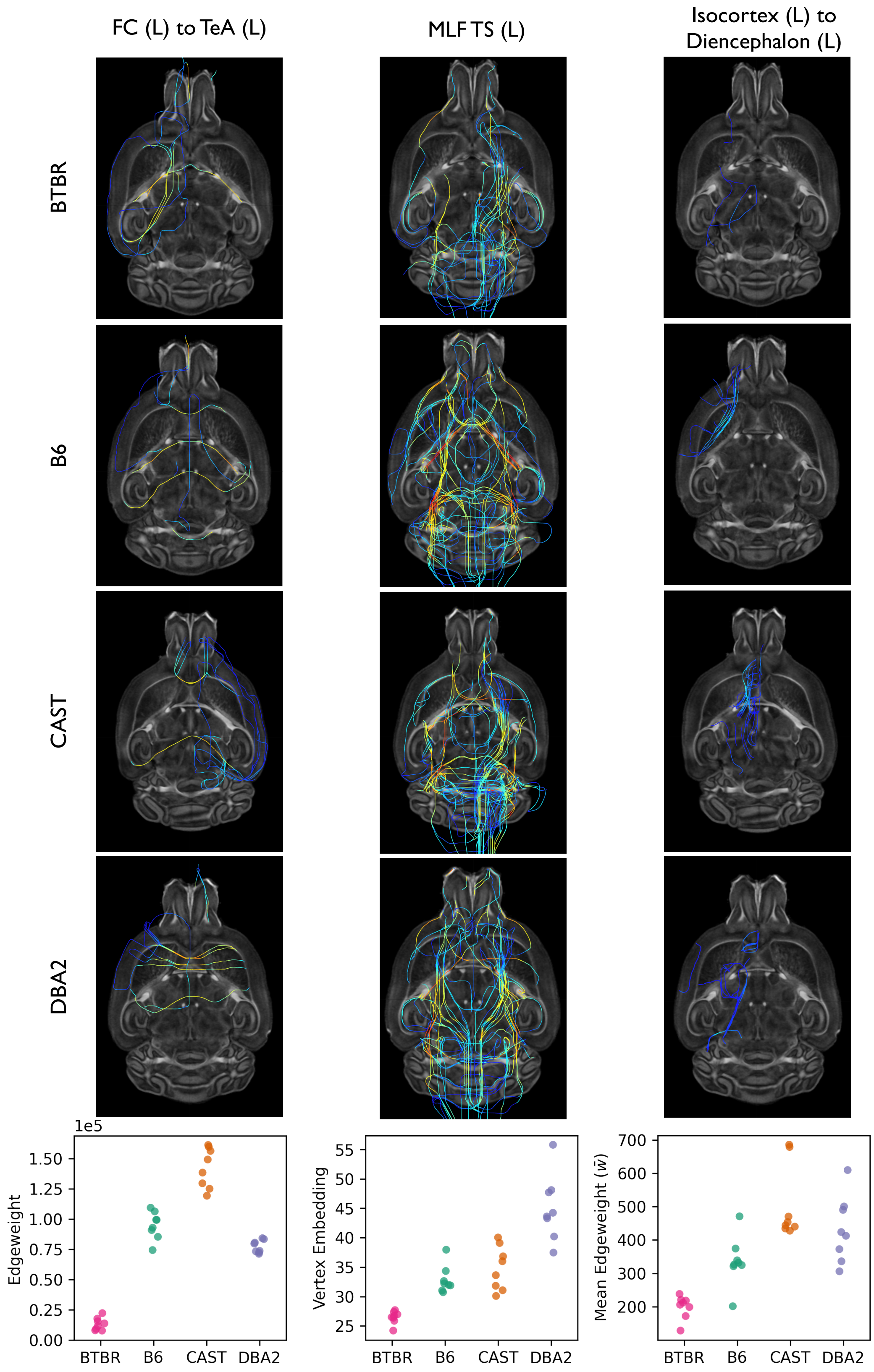

Supplementary Figure 4: Visualization of the weakest signal edge (the left frontal cortex to the right temporal association cortex), vertex (the left medial longitudinal fasciculus and tectospinal tract), and community (left isocortex to left diencephalon) across all mouse strains. At each topological level, tractograms of these neurological structures are shown for each mouse strain. Compared to Figure 3, tractograms here are much more homogeneous across strains, displaying minor differences in connectivity. (*Bottom row*) The distribution of edge weights for the weakest signal edge (*Column 1*); the distribution of the first embedding dimension for the weakest signal vertex (*Column 2*); and the distribution of average edge weight for the most weakest signal community (*Column 3*). Each dot represents data from an individual mouse. Differences in distributions are much less pronounced compared to Figure 3. In these distributions, one strain is typically aberrant while the other three strains are homogeneous.

Vertex	statistic	p-value
Substantia Nigra (L)	0.855	3.28e-05
Middle Cerebellar Peduncle (R)	0.852	3.45e-05
Internal Capsule (L)	0.851	3.5e-05
Substantia Nigra (R)	0.845	3.84e-05
Pontine Reticular Nucleus (R)	0.840	4.14e-05
Pontine Reticular Nucleus (L)	0.838	4.25e-05
Parasubiculum (L)	0.838	4.25e-05
Ventral Tegmental Area (R)	0.837	4.32e-05
Retro Rubral Field (L)	0.829	4.91e-05
Fastigial Medial Nucleus of Cerebellum (R)	0.824	5.3e-05
Cerebral Peduncle (R)	0.819	5.74e-05
Cerebral Peduncle (L)	0.818	5.87e-05
Fastigial Medial Nucleus of Cerebellum (L)	0.814	6.23e-05
Fimbria (L)	0.813	6.28e-05
Brain Stem Rest (L)	0.811	6.46e-05
Cingulate Cortex Area 30 (L)	0.810	6.6e-05
Ventral Tegmental Area (L)	0.806	6.99e-05
Globus Pallidus (R)	0.802	7.46e-05
Internal Capsule (R)	0.797	8.09e-05
Subthalamic Nucleus (R)	0.792	8.78e-05
Zona Incerta (R)	0.783	0.000102
Parabrachial Nucleus (L)	0.783	0.000102
Globus Pallidus (L)	0.781	0.000104
Insular Cortex (R)	0.780	0.000106
Brain Stem Rest (R)	0.777	0.00011
Secondary Visual Cortex Mediolateral Area (R)	0.777	0.00011
Inferior Colliculus (R)	0.776	0.000111
Superior Cerebellar Peduncle (L)	0.775	0.000113
Retro Rubral Field (R)	0.773	0.000117
Stria Terminalis (L)	0.772	0.000118
Postsubiculum (R)	0.769	0.000123
Midbrain Reticular Nucleus (L)	0.768	0.000125
Striatum (R)	0.768	0.000125
Primary Visual Cortex Binocular Area (R)	0.768	0.000125
Corpus Callosum (L)	0.767	0.000125

Table 1. The top 34 signal vertices (out of 326 total vertices) detected by MDMR, ranked by their Holm–Bonferroni corrected p-values. Using MDMR, the corpus callosum is the 34th ranked signal vertex.

	Left Hemisphere		*Right Hemisphere*		
**Vertex**	**p-value**	**Rank**	**p-value**	**Rank**	**Avg. Rank**
Corpus Callosum	5.09e-25	1	1.09e-23	2	1.5
Secondary Motor Cortex	7.73e-20	3	3.1e-16	7	5.0
Internal Capsule	1.6e-14	11	3.26e-16	8	9.5
Stria Terminalis	1.25e-13	18	2.59e-14	12	15.0
Fimbria	1.94e-19	5	3.58e-12	26	15.5
Ventral Tegmental Area	3.87e-12	27	8.76e-13	21	24.0
Hippocampus	8.02e-11	40	7.43e-14	15	27.5
Ectorhinal Cortex	8.11e-12	29	4.56e-11	37	33.0
Globus Pallidus	9.68e-11	41	1.89e-12	25	33.0
Cerebral Peduncle	4.64e-10	57	9.98e-15	10	33.5

Table 2. The top 10 bilateral signal vertex pairs (out of 181 total vertex pairs), as determined by `Omni` and `MANOVA`, ranked by their average ranked p-values.
